# Equilibria and oscillations in cheat–cooperator dynamics

**DOI:** 10.1093/evlett/qrad032

**Published:** 2023-07-22

**Authors:** Ming Liu, Geoff Wild, Stuart A West

**Affiliations:** Department of Biology, University of Oxford, Oxford, United Kingdom; Department of Mathematics, The University of Western Ontario, London, ON, Canada; Department of Biology, University of Oxford, Oxford, United Kingdom

**Keywords:** serial passage, density dependence, frequency dependence, cyclic dynamics, population bottleneck, stochastic group formation

## Abstract

Cooperative societies can be threatened by cheats, who invest less in cooperation and exploit the contributions of others. The impact of cheats depends on the extent to which they are maintained in the population. However, different empirical studies, across organisms ranging from RNA replicators to bacteria, have shown diverse cheat–cooperator dynamics. These vary from approaching a stable equilibrium to dynamic cyclical oscillations. The reason for this variation remains unclear. Here, we develop a theoretical model to identify the factors that determine whether dynamics should tend toward stable equilibria or cyclical oscillations. Our analyses show that (1) a combination of both periodic population bottlenecks and density-dependent selection on cheating is required to produce cyclical oscillations and (2) the extent of frequency-dependent selection for cheating can influence the amplitude of these oscillations but does not lead to oscillations alone. Furthermore, we show that stochastic group formation (demographic stochasticity) can generate different forms of oscillation, over a longer time scale, across growth cycles. Our results provide experimentally testable hypotheses for the processes underlying cheat–cooperator dynamics.

## Introduction

**Figure 1.**
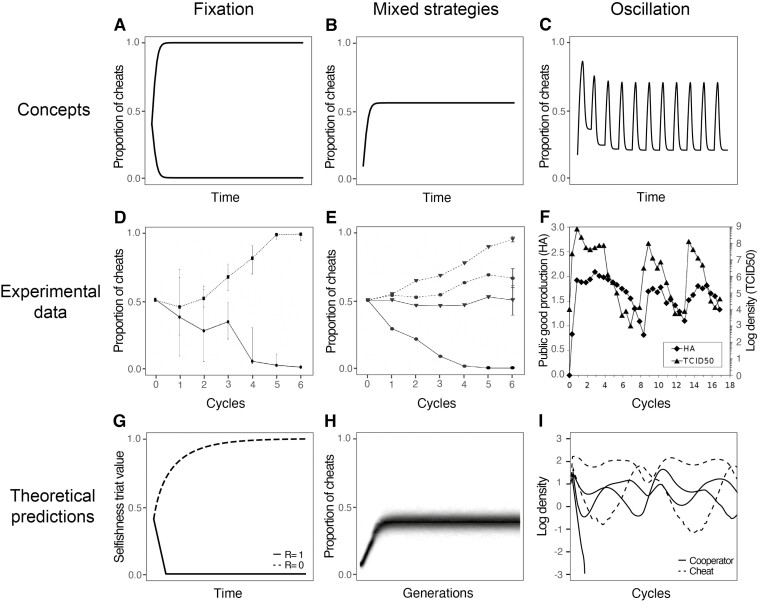
Different types of cheat–cooperator dynamics. (A–C) Conceptual illustrations of different cheat–cooperator dynamics: (A) either cheats or cooperators go to fixation; (B) cooperators and cheats are maintained at an equilibrium proportion in a population (coexistence); (C) oscillations through time. (D–F) Empirical examples of different cheat–cooperator dynamics: (D) proportion of cheats that do not quorum sense in the bacteria *P. aeruginosa* (Rumbaugh et al., 2012); (E) proportion of cheats that does not produce siderophores in the bacteria *P. aeruginosa* (Griffin et al., 2004); (F) production of public goods by influenza virus and its total population density (Frensing et al., 2013). (G–I) Previous theoretical examples of different cheat–cooperator dynamics: (G) Taylor and Frank (1996); (H) Killingback et al. (2010); (I) (Mizuuchi et al. (2022).

All populations of cooperative organisms are at risk of being invaded by cheats, defined as individuals who avoid the costs of cooperation but benefit from the cooperation of others (Ghoul et al., 2014; Trivers, 1971). Cheats have been observed at many levels of biology, from genes to viruses, and bacteria to animals (Ghoul & Mitri, 2016; He et al., 2019; Hughes & Boomsma, 2008; Jaenike, 2001; Strassmann & Queller, 2011; Tapia et al., 2019). In some cases, cheats are individuals who do not produce a public good, such as replicase enzyme in viruses or iron-scavenging molecules in bacteria (Griffin et al., 2004; Leeks et al., 2021). In other cases, cheating can take more active or devious forms, such as when a cuckoo or other brood parasite tricks individuals of another species into rearing their eggs (Davies, 2015). In the extreme, if cheats spread to fixation, then cooperation can be lost (Andersen et al., 2015). Consequently, the influence of cheats will depend on their evolutionary dynamics. Will cheating be transient, spread to an equilibrium level, go to fixation, or should we even expect cycles of cheating (Fig. 1A–C)?

Very different cheat dynamics have been observed across empirical studies. In many laboratory experiments with bacteria the dynamics approach an equilibrium, where cheats are either lost, go to fixation, or maintained at a relatively stable proportion (i.e., frequency; Figure 1D and E) (Diggle et al., 2007; Frost et al., 2018; Griffin et al., 2004; Pollitt et al., 2014; Rumbaugh et al., 2012; Schluter et al., 2016). In contrast, laboratory experiments with RNA replicators and data from natural populations of bacteria have shown cheats cyclically oscillating in proportion (Furubayashi et al., 2020; Kümmerli et al., 2015; Mizuuchi et al., 2022; Raymond et al., 2012). Cyclical oscillations in the proportion of cheats are also observed when culturing viruses (Frensing et al., 2013; Tapia et al., 2019). Indeed, these cycles are so common across different virus species that they have been given a name—the von Magnus effect (Kirkwood & Bangham, 1994; Tapia et al., 2019; von Magnus, 1951; Zwart et al., 2013) (Figure 1F). It is not clear why the dynamics of cheats varies so much across these empirical studies. Why do cheats show stable dynamics in some studies, but oscillate in proportion in others?

The dynamics of cheating is also important for assessing the validity of some intervention strategies for treating parasites. Synthetic viral cheats, termed “therapeutic interfering particles,” are being developed to interfere with and suppress viral infections (Dimmock & Easton, 2014; Leeks et al., 2021; Metzger et al., 2011). Similar strategies have been suggested for bacteria, using cheats to introduce either less virulent strains or medically beneficial alleles such as antibiotic susceptibility into infections (Brown et al., 2009). It has also been shown both theoretically and experimentally that it can be harder for parasites to evolve resistance against intervention strategies that disrupt cooperation, than against treatments such as antibiotic treatments that kill individuals (André & Godelle, 2005; Dieltjens et al., 2020). Disrupting cooperation turns all individuals into cheats, making it hard for a “resistance” mutation that switches cooperation back on to spread (Mellbye & Schuster, 2011). In all of these cases, the effectiveness of the intervention will depend upon the cheat–cooperator dynamics. For example, if the proportion of cheats can oscillate in ways that allow the cheats to go extinct, then the intervention will be less useful (Rezzoagli et al., 2019).

Theoretical models have made contrasting predictions for the evolutionary dynamics of cheating. Hamilton found that the fitness of cheats did not depend on their proportion in the population, and so cheats would either be disfavored or go to fixation (Figure 1G) (Hamilton, 1964). More recent studies have found that the fitness of cheats can be greater when they are less common (frequency dependence) or when populations are more dense (density dependence) (Gore et al., 2009; MacLean & Gudelj, 2006; Madgwick et al., 2018; Patel et al., 2019; Queller, 1984; Ross-Gillespie et al., 2007; 2009; Scholz & Greenberg, 2015; van Gestel et al., 2014). These processes can result in both cooperators and cheats being maintained at some equilibrium proportion in a population (coexistence; Figure 1H). Other models have predicted nonequilibrium dynamics, in which the frequencies of cheats and cooperators oscillate over time (Figure 1I) (Mizuuchi et al., 2022; Sanchez & Gore, 2013; Weitz et al., 2016). Because many of these models were tailored to specific experimental systems and contained multiple different features, it is not clear why they give rise to different predictions. Why do we get stable equilibrium with some models, versus oscillations in cheat proportion in others?

We investigate theoretically how different life-history features can interact to produce different forms of cheat–cooperator dynamics. We use a combination of numerical and simulation approaches to examine the influence of four factors that could shape the dynamics of cheating: frequency dependence, density dependence, periodic population bottlenecks, and stochastic group formation. These factors have been suggested to be important in previous theories but never examined together. Our main aims are to (1) understand what maintains oscillations in the proportion of cheats and cooperators, as opposed to a tendency toward equilibrium; (2) use the results of our model to explain variation across previous theoretical and empirical studies; and (3) provide experimental designs for testing the empirical influence of different factors on cheat–cooperator dynamics.

## Methods and results

We developed a cheat–cooperator model that allows for several factors that could potentially alter the form of dynamics. We investigated how the proportion of cheat is influenced by frequency and density dependence on cooperators, periodic population bottlenecks, and stochastic group formation. Frequency dependence is when cheats’ growth rate depends on the proportion of cooperators within population; density dependence is when it depends on the number of cooperators. By changing the parameters of our model, we could include or remove these different factors, and therefore test their different influences as well as their interactions.

### The model

We derived our model from competitive Lotka–Volterra equations where a system of differential equations described the change in population density over infinitesimally small increments of time:


$$\begin{array}{l} \left\{ \begin{array}{c} \frac{dN_{co}}{dt}=rN_{co}\left(1-\frac{N_{co}+\alpha N_{ch}}{K}\right)\;\frac{dN_{ch}}{dt}=hrN_{ch}\left(1-\frac{\alpha N_{co}+N_{ch}}{K}\right)\; \end{array}\right., \end{array}$$ (1)

where $N_{co}$ and $N_{ch}$ are the density of cooperator and cheats, *r* is the intrinsic growth rate, *α* is the relative strength of between-strain population regulation to within-strain regulation, and *K* is the carrying capacity of the system (Lotka, 1925; Volterra, 1927). Equation 1 is comparable to a population with a limited supply of nutrients that stops growing once the nutrient has been depleted (i.e., the population reaches its carrying capacity). We simplified the interactions between populations so that cooperators and cheats have the same regulation on each other (i.e., *α* = 1). The growth rate of cheats is the same, except for the benefit coefficient of cheating (*h*). The benefit coefficient rescales the growth rate of cheats: If *h* < 1, cheats can never grow as fast as cooperators; if *h* ≥ 1, cheats can grow equally well or better than cooperators.

We then extended equation 1 to examine the consequences of when population density and the frequency of cheats influence the relative fitness of cheats. We assumed:


$$\begin{array}{l} \left\{ \begin{array}{c} \frac{dN_{co}}{dt}=rN_{co}\left(1-\frac{N_{co}+\alpha N_{ch}}{K}\right)\;\frac{dN_{ch}}{dt}=\left(1-a+\frac{a}{1+e^{-s_{d}\left(N_{co}-t_{d}\right)}}\right)\left(1-b+\frac{b}{1+e^{-s_{f}\left(N_{co}/\left(N_{co}+N_{ch}\right)-t_{f}\right)}}\right)hrN_{ch}\left(1-\frac{\alpha N_{co}+N_{ch}}{K}\right)\;\; \end{array}\right., \end{array}$$ (2)

where two additional terms are multiplied to the rate of changes in the cheat density: the density dependence term in blue texts and the frequency dependence term in orange text. The cheat density dependence term $\left(1-a+\frac{a}{1+e^{-s_{d}\left(N_{co}-t_{d}\right)}}\right)$ describes how the relative fitness of cheats increases at higher population densities because they are better able to exploit cooperators. For example, when bacteria are at higher densities, cheats will on average be closer to cooperators and hence better able to exploit cooperative behaviors, such as the production of public goods molecules (Kümmerli et al., 2009; Ross-Gillespie et al., 2009). The importance of cheat density dependence is weighted by the parameter *a*, where *a* = 0 means that there is no density dependence. The shape parameter $\left(s_{d}\right)$ controls the steepness of the density dependence function by changing the function from a binary response (higher $s_{d}$) to a gradual transition (lower $s_{d}$). The threshold parameter $\left(t_{d}\right)$ controls the location of the density dependence function, where a high threshold means the transition in cheats’ growth rate occurs at a higher density of cooperators. This way in which the relative fitness of cheats can vary with density (cheat density dependence) is different and needs to be distinguished from how population growth decreases as the population density approaches the carrying capacity (population density regulation, $1-\frac{N_{co}+N_{ch}}{K}$). In Supplementary Section 3.1 and Supplementary Figure S3, we provide a graphical explanation of these model parameters.

The cheat frequency dependence term $\left(1-b+\frac{b}{1+e^{-s_{f}\left(N_{co}/\left(N_{co}+N_{ch}\right)-t_{f}\right)}}\right)$ describes how the relative fitness of cheats increases when they at lower frequencies (proportion) in the population because they are better able to exploit cheats. For example, when bacteria or yeast cheats are a lower fraction in the population then they are more likely to be interacting with cooperators (Ross-Gillespie et al., 2007; Gore et al., 2009). The importance of cheat frequency dependence is weighted by the parameter *b*, where *b* = 0 means that there is no frequency dependence. The shape $\left(s_{f}\right)$ and threshold $\left(t_{f}\right)$ parameters determine the steepness and location of the frequency dependence function.

The focus of our result will be on the effects of weightings of density and frequency dependence (i.e., *a* and *b*) because the difference in growth rate between cheats and cooperators is the product of (1) the density dependence term; (2) the frequency dependence term; and (3) the benefit coefficient of cheating (*h*). As the density and frequency of cooperators can vary through time, the relative benefit of cheating can also have temporal fluctuation. All parameter values are summarized in Supplementary Table S1 and discussed further in Supplementary Information.

### Scenario 1: Frequency and density dependence

We started by examining an unstructured and well-mixed (panmictic) population, without population bottlenecks or stochastic group formation. Biologically speaking, scenario 1 is analogous to chemostats in experimental evolution, where abiotic factors are kept constant through time (Gresham & Dunham, 2014; Novick & Szilard, 1950).

### Result 1: Density and frequency dependence are insufficient for dynamic oscillations

**Figure 2.**
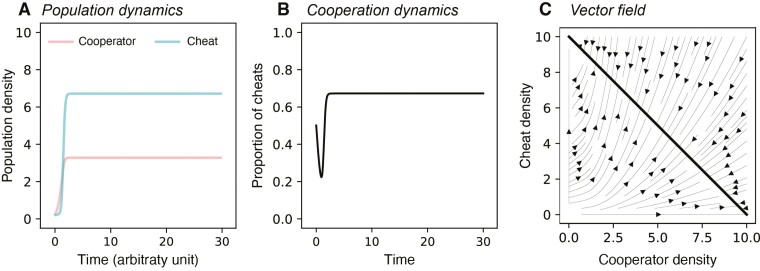
Dynamics of the model in a simple set up. (A) Density of cooperator and cheat through time (i.e., population dynamics; $N_{co}$ and $N_{ch}$). (B) Proportion of cheats through time (i.e., cooperation dynamics; $N_{ch}/(N_{co}+N_{ch})$). (C) Vector field plot showing the model would have static dynamics once population densities reach carrying capacity (shown in thick black line) (*a* = 1, *b* = 0; other parameter values are listed in Supplementary Table S1).

By independently varying the levels of density (0≤a≤1) and frequency (0≤b≤1) dependence, we found that neither density nor frequency dependence led to dynamic oscillations. Here we are referring to density dependence in terms of how density influences the relative fitness of cheats (equation 2), and not how the overall population density influences the rate of growth towards the carrying capacity (equation 1). In all cases, we found that our model approached an equilibrium, with the coexistence of cooperators and cheats at a certain ratio (Figure 2A and B; analytical analysis in Supplementary Section 2.2).

This model reaches equilibria because the population density regulation term, $1-\frac{N_{co}+N_{ch}}{K}$, leads to the populations of both cooperators and cheats remaining fixed once the carrying capacity is reached $\left(N_{co}+N_{ch}=K\right)$. We can also examine the dynamics via a vector plot, which shows the direction of changes in population densities from any state (Otto & Day, 2007). In other words, the arrows in a vector plot would indicate how the population dynamic should change in the two-dimension parameter space of the densities of cheats and cooperators. Our vector plot confirms our predictions, by showing that all dynamics converge to the equilibrium point (line) where $N_{co}+N_{ch}=10$ (Figure 2C; thick black line). The level of frequency dependence (*b*) can alter the equilibrium proportion of cheats, but it does not change the form of dynamics from approach to an equilibrium (Supplementary Section 2.1). These results are consistent with a number of previous theoretical studies have shown how density or frequency dependence can lead to coexistence between cheats and cooperators at some equilibrium frequency (Gore et al., 2009; MacLean & Gudelj, 2006; Patel et al., 2019; Queller, 1984; Ross-Gillespie et al., 2007; 2009).

### Scenario 2: Periodic population bottlenecks

We then relaxed the assumption that the population is free from disturbance by assuming it goes through periodic bottlenecks. We dilute the population densities of both cheats and cooperators by a dilution ratio, D, once every $T_{grow}$ time units. This leads to the population dynamics following the algorithm of


$$\begin{array}{l} \left\{ \begin{array}{c} if\;t<T_{grow}:equation\;1,\;t=t+dt\;if\;t=T_{grow}:\;N_{ch}=N_{ch}/D,\;N_{co}=N_{co}/D,t=0\;\; \end{array}\right., \end{array}$$ (3)

where $T_{grow}$ is the duration of each growth period. Biologically speaking, expression 3 is analogous to serial passages, or growth cycles and host–host transmission, in experimental evolution (Gresham & Dunham, 2014; Lenski et al., 1991). Some theoretical studies have discussed the optimal dilution ratio for experimental evolution, but density or frequency dependence was not included (Wahl & Gerrish, 2001; Wahl et al., 2002). Another study has looked into cooperation dynamics under different bottleneck settings, but without temporal dynamics (Brockhurst et al., 2007).

### Result 2: The combination of periodic population bottlenecks and density dependence generates cheat–cooperator oscillations

**Figure 3.**
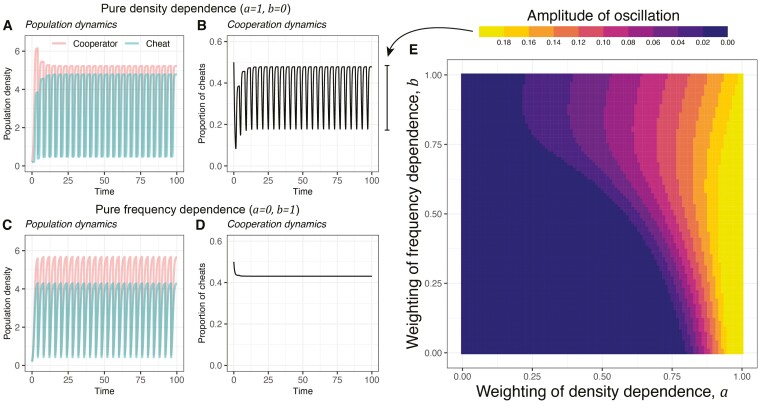
Density dependence (*a* > 0) is crucial for oscillation in frequency dynamics. (A, B) Density and frequency dynamics when density dependence of cheats on cooperators is at the strongest level and frequency dependence is absent (*a* = 1, *b* = 0). (C, D) Dynamics when density dependence is absent and frequency dependence is strongest (*a* = 0, *b* = 1). (E) Broader exploration of amplitude in oscillation, which is defined as the maximal difference in proportion of cheats, across all combinations of density and frequency dependence weighting.

We found that the combination of periodic population bottlenecks and cheat density dependence could lead to dynamic cycles, where the proportion of cheats and cooperators oscillates over time, rather than approaching an equilibrium (e.g., *a* = 1, *b* = 0, *D* = 10; Figure 3A and B). This combination leads to oscillations because the population bottleneck moves populations from high density, where cheats are increasing in proportion, to low density, where cooperators increase in proportion. It is this change in whether cooperators or cheats are preferentially favored that keeps the oscillations going. After a bottleneck, cooperators are favored and so increase in proportion. Then as the population grows, the relative fitness of cheats increases, until eventually cheats have a higher fitness and then increase in proportion (Supplementary Section 3.5).

In contrast, the combination of population bottlenecks and frequency dependence did not lead to dynamic oscillations (e.g., *a* = 0, *b* = 1; Figure 3C and D). This combination does not lead to oscillations because the population bottleneck did not change whether cheats or cooperators were favored. If cheats are initially introduced at a low proportion, and have a fitness advantage, they will then increase in proportion, causing their relative fitness to decrease. Eventually, the proportion is reached at which the fitness of cheats and cooperators is equal, and so an equilibrium is reached. Population bottlenecks do not change this qualitative pattern because they change density not frequency. Consequently, bottlenecks do not lead to a scenario where cooperators are at an advantage, which would require a change in the proportion of cheats, rather than just the population density.

Additionally, the extent of frequency dependence (*b*) can interact with the extent of density dependence (*a*) to determine the amplitude of cyclic oscillations (Figure 3E). The oscillation amplitude is the maximum difference in proportion of cheats once the dynamics have stabilized (the bar next to Figure 3B). The amplitude of oscillation increases with increasing cheat density dependence (colors become brighter as one moves from left to right). Frequency dependence has mixed effects on amplitude of oscillation, depending on the weighting of density dependence—increasing oscillation when density dependence is low, but reducing oscillations when density weighting is high (a≈1). This complex pattern happens because frequency dependence can change the differences in relative fitness of cheats, and if the differences become larger, the amplitude of oscillation can grow.

We examined the effects of all model parameters on the amplitude of oscillation in supplementary information. In Supplementary Sections 3.4 and 3.5, we showed that increasing the benefit of cheating (h, h>1) decreases the range where oscillation happens because cheats can spread even at lower densities. There is no oscillation in cheat proportion when h=1 because cheats can never out-grow cooperators. In Supplementary Section 3.6, we showed that decreasing the dilution ratio (*D*) reduces the amplitude of oscillation. Supplementary Section 3.7 suggested sufficient growth time $\left(T_{grow}\right)$ is needed to generate oscillating dynamics. In Supplementary Section 3.8, we changed the values of shape parameters and found steeper density dependence functions (larger $s_{d}$) increase the amplitude of oscillation, while steeper frequency dependence functions (larger $s_{f}$) reduce the optimal frequency weighting (*b*) where oscillation is favored. In Supplementary Section 3.9, a higher threshold of density dependence function (larger $t_{d}$) reduces the range of density weighting (*a*) where cheat proportion oscillates; a higher threshold of frequency dependence function (larger $t_{f}$) also reduces the optimal frequency weighting (*b*) for oscillation. Lastly, we showed our setting of ODE solver and intrinsic growth rate is robust against periodic population bottlenecks (Supplementary Sections 3.2 and 3.3).

### Scenario 3: Stochastic group formation

Finally, we relaxed the assumption of unstructured and panmictic populations. We examined the stochastic consequences of small populations or incomplete population mixing processes (Constable et al., 2016; Hilbe et al., 2018; Mizuuchi et al., 2022). We could not examine the role of stochasticity with our nonlinear deterministic population dynamical model (equation 2), and so we instead developed an individual-based simulation. We modeled a population growing at the unit of subpopulations, where each subpopulation is partially mixed before going through periodic population bottleneck, following Mizuuchi et al. (2022). However, we simplified their system to only two possible strains (one cooperator strain and one cheat strain) and replaced the ordinary differential equations with equation 2 to model population growth of cooperators and cheats with density and/or frequency-dependent benefits.

Our simulation divides the population into *M* subpopulations (social groups), where the interactions between cheats and cooperators take place. The life cycle then involves two phases. First, a growth phase in the subpopulations. Second, a phase of subpopulation mixing, before samples (bottlenecks) are taken to initiate the next round of subpopulation growth. Population mixing is only partial between subpopulations, with each new subpopulation being formed from a sample of just two subpopulations, rather than all subpopulations. This design helps slow down the spread of cheats or cooperators. We repeated the random sampling of two subpopulations and creation of two new subpopulations for F×M times to start the next growth cycle, where F is the mixing coefficient (i.e., larger *F* means more mixing process and closer to global mixing). Following the experimental protocols and algorithms by Mizuuchi et al. (2022), we converted the population densities to integers when they were sampled, summed, and after diluted. This process increases stochasticity in finite populations and has been found to help generate oscillating dynamics (Mizuuchi et al., 2022). A full list of parameters is listed in Supplementary Table S1 and the simulation process is illustrated in Supplementary Section 4.1.

Our simulation allows us to investigate whether stochasticity influences cheat–cooperator dynamics by varying the number of subpopulations (*M*), the carrying capacity of each subpopulation (*K*), and the mixing coefficient (*F*). These three parameters have experimental analogues and can change stochasticity in different ways. Increasing the number of subpopulations (*M*) increases the size of the entire population, thus increases the sample size of random sampling and initializing new subpopulations, and decreases stochasticity. Increasing carrying capacity (*K*) increases the number of individuals available to be distributed into new subpopulations, thus decreases the stochasticity in each process. Increasing the mixing coefficient (*F*) can reduce stochasticity by making each subpopulation more similar to each other, but increased mixing also disrupts the population structure and facilitates the spread of cheats or cooperators to other groups.

### Result 3: Stochasticity can also generate cheat–cooperator oscillations

**Figure 4.**
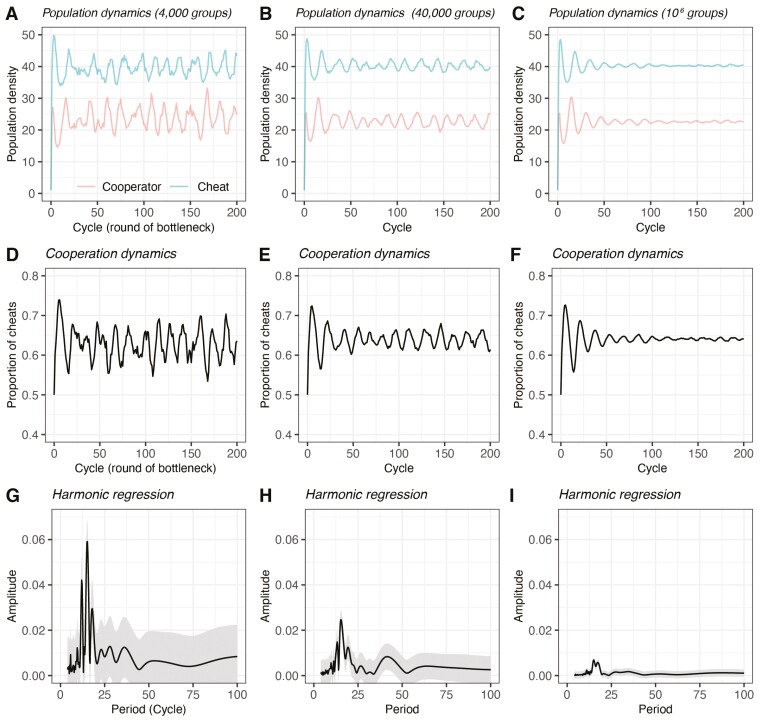
Stochasticity creates an additional layer of oscillation across growth cycles. (A–C) Population dynamics at various degree of stochasticities, where increasing number of subpopulations results in an increased global population size, thus decreasing stochasticity (because the carrying capacity of each subpopulation is the same). (D–F) Cooperation dynamics of the density time series in (A–C). (G–I) Harmonic regression analyzed the time series of cheat frequencies from cycle 51 to cycle 200 and showed the estimated amplitude at each period with confidence intervals. Periodicity of cheat frequencies increases if certain periods have much higher amplitude than other periods (e.g., G and H compared to I). The results presented here are density-dependence-only model, where *a* = 1 and *b* = 0.

We found that stochasticity, combined with density dependence and population bottlenecks, can lead to oscillations in the proportion of cheats across cyclic population bottlenecks (e.g., *a* = 1, *b* = 0; Figure 4A and D). This oscillation is different from result 2, where the oscillation has identical dynamics in each growth cycle; instead, the oscillation here occurs across (rather than within) bottlenecks and is somewhat noisier (i.e., Figure 4 quantifies time at the unit of growth cycle, where each grow cycle lasts $T_{grow}$). We quantified the cyclic nature of these oscillations with harmonic regression (Halberg et al., 1967; Young et al., 1999), which examines the extent to which sine waves can explain the observed time series data. The results showed a peak of amplitudes at 10–20 growth cycles (Figure 4G), meaning that cheat proportion roughly repeats the same oscillating dynamic every 10–20 growth cycles under the focal parameter setting. In addition, we investigated the role of stochasticity by examining how these oscillating dynamics disappear as stochasticity is reduced. The cyclic oscillation becomes smaller with intermediate number of subpopulations (Figure 4B and E) and quickly attenuated in large number of subpopulations (Figure 4C and F). Harmonic regression further showed that the size of peak was smaller when there were more subpopulations (Figure 4G–I). This pattern occurs because increasing sample size results in a less stochastic distribution of cheat proportion across all subpopulations, as suggested by central limit theorem. Similarly, by increasing the number of individuals in each subpopulation, we found increasing carrying capacity (*K*) also produces smaller oscillation in Supplementary Section 4.2.

**Figure 5.**
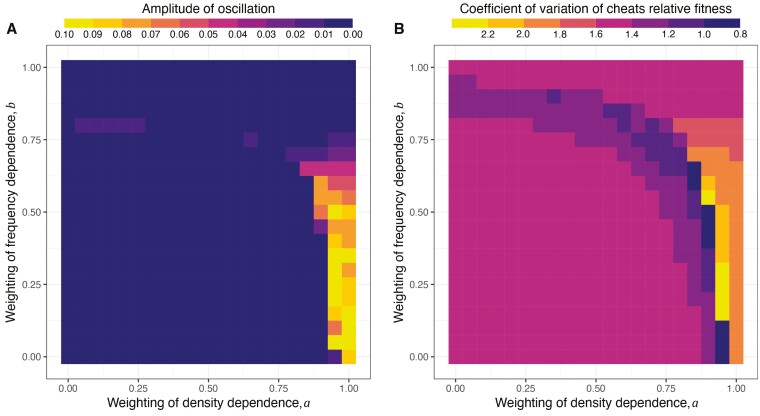
The impacts of density and frequency dependence on oscillations across growth cycles and fitness landscape in scenario 3. (A) Amplitude of oscillation is calculated from the maximal signal of cheat proportion after discrete Fourier transformation. Brighter colors represent greater oscillation. (B) Coefficient of variation of relative fitness of cheats is calculated from the relative fitness of all subpopulations, using standard deviation divided by average, for each growth cycle. In all panels, each grid is the average of 10 repeated simulations. All simulations last 200 growth cycles and data was collected from cycle 51 to cycle 200. The number of subpopulations is set to 4,000.

In order to understand the cause of oscillating dynamics in cheat proportion in scenario 3, we continued our analysis to a broader scale of density and frequency dependence. We analyzed three properties: the amplitude of oscillation in cheat proportion, where larger values mean the cooperation dynamics are more variable or periodic (Figure 5A); cheat’s average relative fitness, where values greater than 1 indicate cheats are fitter than cooperators (Supplementary Section 4.3 and Supplementary Figure S17); and the coefficient of variation, where larger values suggest relative fitness is more variable between subpopulations (Figure 5B). These factors are potentially important because they inform us about the prevalence of oscillations, and the fitness landscape across different scenarios. We found strong density dependence and weak-to-moderate frequency dependence can generate oscillating dynamics (*a* > 0.8, *b* < 0.7). Under these combinations of density and frequency dependence, cheats have relative fitness between 1 and 1.5, which means cheats can slightly outcompete cooperators on average. Furthermore, these combinations produce the highest coefficient of variation across the entire parameter space. This finding coincides with previous work on synthetic microbial system, where large variation was generated through strong population bottlenecks (Chuang et al., 2009). In short, we found that oscillating dynamics in cheat proportion is associated with small fitness advantage of cheats over cooperators, and high variation of fitness between subpopulations.

Why does stochasticity lead to oscillations? We found that oscillations occurred when there is greater variations in the fitness of cheats, such that cheats have greater fitness than cooperators in some patches but lower in others (Figure 5B). We hypothesized that this fitness variation can lead to cycles, and not just noise, because limited mixing between groups delays the rates where a group can reach an equilibrium proportion of cheats. We tested this hypothesis by changing the degree of mixing between groups. In support of our hypothesis, we found that greater mixing produces a less periodic dynamic—as the mixing coefficient (*F*) increases, the peak signal in harmonic regression becomes smaller, while the nonpeak signals become larger (Supplementary Section 4.4 and Supplementary Figure S18, but see Supplementary Section 4.5 for a special exception).

## Discussion

Our model predicted three different forms of cheat–cooperator dynamics: approach to a stable proportion of cheats (Figure 2); cyclic oscillations of the proportion cheats within growth cycles (Figure 3); and cyclic oscillations of the proportion cheats across growth cycles (Figure 4). A combination of both periodic population bottlenecks (growth cycles or serial passages) and density dependence was required to produce cyclic oscillations which repeat each growth cycles (Figure 3E). The stochasticity introduced by small populations or small number of subpopulations was required to produce oscillations in the proportion of cheats across growth cycles (Figure 4 and Supplementary Section S16).

Our model can explain the variation in predictions between previous theoretical models (Table 1). First, comparing with our scenario 1, models that assumed frequency or density dependence, but without periodic population bottlenecks, predicted evolution toward an equilibrium proportion of cheats (Brown et al., 2009; Patel et al., 2019; Ross‐Gillespie et al., 2007, 2009). Second, comparing with our scenario 2, models that assumed both periodic population growth, and cheat density dependence, predicted oscillations within growth cycles (Sanchez & Gore, 2013; Yurtsev et al., 2013). Third, comparing with our scenario 3, models that allowed for stochasticity, as well as periodic growth cycles and cheat density dependence, led to oscillations across growth cycles (Mizuuchi et al., 2022; Vetsigian, 2017). Brown and Taddei (2007) found a different mechanism of oscillation through durable public goods which remains exploitable for several generations. Because the dynamics of public goods is slower than cheat proportion, there is a delay in the consequence of current players’ strategies, which results in cyclic dynamics. Similarly, Weitz et al. (2016) predict oscillations for another different reason. In their model, cheats initially have an advantage, but degrade the environment, till a switch point is reached at which point cooperators have an advantage. As cooperators become more common, the environment improves, until the switch at which cheats are again favored. This leads to oscillations because a switch point is assumed, which swaps which strategy has an advantage—as opposed to the more gradual functions in our scenario 1, which allowed an equilibrium to be reached.

**Table 1.** The list of published theory papers.

Paper	Focus	Oscillate?	Experiment?	Equivalent
Eco-evolutionary models				
Sanchez and Gore (2013)	Cooperation dynamics	Yes (within cycle)	Yes	Density dependence + growth cycle
Yurtsev et al. (2013)	Cooperation dynamics under antibiotic stress	Yes (within cycle)	Yes	Density dependence + growth cycle
Vetsigian (2017)	Coexistence of strains with different toxin production and resistance	Yes	No	Density dependence + growth cycle + stochasticity
Mizuuchi et al. (2022)	Cooperation dynamics	Yes	Yes	Density dependence + growth cycle + stochasticity
Replicator dynamics				
Brown and Taddei (2007)	Cooperation dynamics with durable public goods	Yes	No	N/A
Weitz et al. (2016)	Cooperation dynamics with game–environment feedback	Yes	No	N/A

Our model can help explain the variation in cheat–cooperator dynamics that have been observed across previous empirical studies. First, oscillations have been observed in a long-term experimental evolution study of synthesized cooperative and cheat (defective) RNA replicators (Furubayashi et al., 2020; Mizuuchi et al., 2022). This system appears to fit the assumptions of our scenario 3, where we would predict oscillating dynamics across periodic growth cycles. In their experiment, a periodic population bottleneck was imposed, both types of replicators can reproduce during coinfection, and the limiting factor for reproduction is the population size of host bacterial cells. Second, oscillations between cooperators and cheats (defective interfering particles) are common when culturing viruses (Frensing et al., 2013; Grabau & Holland, 1982; Huang, 1973; Palma & Huang, 1974; Roux et al., 1991; Tapia et al., 2019; von Magnus, 1951). This culturing of viruses involves growth cycles with cheat density dependence, and so is analogous to our scenario 2. Several theoretical models have examined these dynamics within predator–prey framework (Bangham & Kirkwood, 1990; Frank, 2000; Heldt et al., 2013; Kirkwood & Bangham, 1994; Shirogane et al., 2021). Predator–prey equations approximate defective interfering particles because there can be a very high benefit to cheating (high *h*), such that cheats can effectively eliminate (eat) cooperators when they coinfect a cell (Leeks et al., 2021). These models provide another mechanism for oscillating dynamics under a more specific biological setting. Third, oscillations of cheat proportion have also been observed in natural populations of the bacteria *Bacillus thuringiensis*, where the life cycle is consistent with our scenario 2: There is appreciable density dependence and winter imposes a periodic population bottleneck each year (Raymond et al., 2012).

There are also several experimental evolution studies in bacteria and viruses, where cyclical oscillations were not observed (Diggle et al., 2007; Frost et al., 2018; Griffin et al., 2004; Meir et al., 2020; Özkaya et al., 2018; Pollitt et al., 2014; Rumbaugh et al., 2012; Turner & Chao, 1999). The dynamics by which stable equilibria were reached in those cases suggest that cheat density dependence was not strong enough to change which strategy had the higher fitness, after the periodic bottleneck. One possible explanation for this is that the strength of density dependence can be lower in the well-mixed liquid populations that are routinely used in such experiments (Kümmerli et al., 2009; Ross‐Gillespie et al., 2009). This and other possibilities could be tested experimentally (see below). Even with periodic population bottlenecks and cheat density dependence, our model only predicts cyclic oscillations under certain parameter conditions—not at all times.

**Figure 6.**
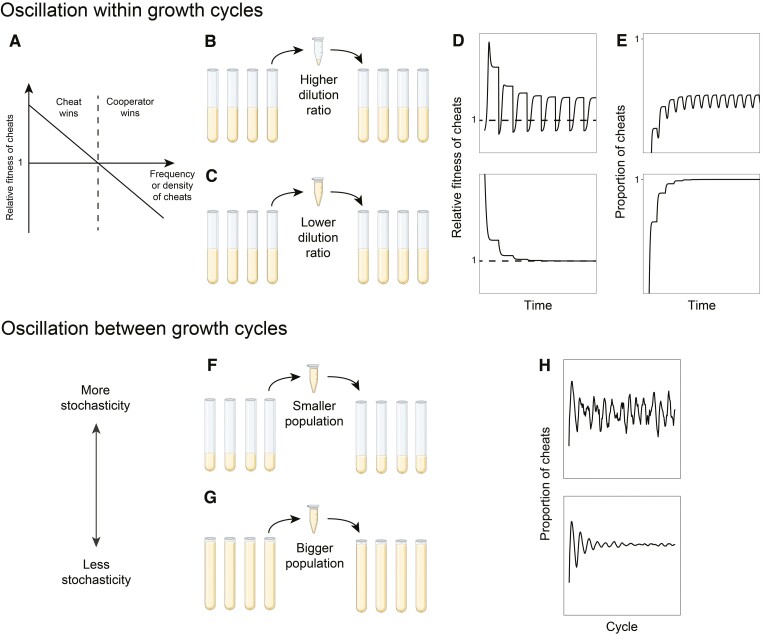
Possible experimental designs for testing the role of different factors in leading to oscillations. (A–E) Changing dilution ratio may create chances for relative fitness of cheats to cross horizontal line of equal fitness (e.g., dashed line in D), and let the direction of selection alternate within each growth cycle. The direction of selection would only change when the relative fitness of cheats crosses unity (dashed line), so that selection sometimes favors cooperators and other times favor cheats. (F–H) Increasing population size could result in a decrease in stochasticity and stop oscillation. Created with BioRender.com.

Our model suggests numerous experimental designs that could be used to test how different factors influence cheat–cooperator dynamics. Considering oscillations within growth cycles, the degree of population bottleneck or strength of density dependence could be reduced, to test whether this eliminates cycles (Figure 6A–E). For example, by diluting populations less each growth cycle, or by adding less nutrients, to restrict growth to low population densities. Another possibility is to parametrize the strength of density dependence, to test whether it is low or lower in studies where cyclic oscillations have not been observed. An even more extreme manipulation would be to compare populations in chemostats, to populations with periodic growth cycles. As for oscillation across periodic growth cycles, the degree of stochasticity could be reduced to examine whether it eliminates oscillations (Figure 6F–H). For instance, by increasing the total volume of medium, or by providing more nutrients, to let the population grow to a larger size. Our results predict oscillation would diminish when stochasticity is lower. Nevertheless, our plan would require enough data points to allow time series tools (e.g., harmonic regression, discrete Fourier transformation, and equivalent autoregressive tools) to distinguish periodic oscillation from pure noise. Our model could also be applied to data from macro-organisms, which experience growth cycles through seasonality.

The theoretical framework developed in this article is flexible and can be easily extended to consider other biological scenarios. For instance, spatial structure can lead to the coexistence of cheats and cooperator (Nowak & May, 1992)—this could be incorporated by assuming explicit spatial arrangements between each patch or subpopulation (with social groups located next to one another). Spatial structure could help generate oscillations because individuals cannot disperse to distant patches, and so some cooperators will be less likely to encounter and hence be exploited by cheats. This is analogous to the stochastic scenario that we investigated in our individual-based simulation (Figures 4 and 5 and Supplementary Figure 18). Spatial structure provides an even more extreme case of low mixing. Another possibility is temporal variation in environmental quality, which can be implemented by varying carrying capacity through time. One benefit of using the current framework is that competitive Lotka–Volterra equations relax the assumptions of constant population size and constant degree of generation overlap. Relaxing these two assumptions has been found to support the selection of specialist against generalist (Gilchrist, 1995; Liu et al., 2019) and the coexistence between competing strains (Huang et al., 2015; Liu et al., 2021). Nevertheless, competitive Lotka–Volterra equations always predict coexistence of strategies when there is no external disturbance, such as noise, growth cycles, or temporal change (scenario 1). Future work could examine scenarios where this is not the case.

Our model could also be modified to examine other types of interaction (Jones et al., 2009; Lion, 2018). We examined a relatively simple cheat–cooperator system. One possible extension is to consider symbiotic or pathogenic bacteria, where being a cheat or cooperator has a large influence on the interaction with their host and could therefore even alter the host dynamics. This could be examined with a three-level host-cooperator-cheat model. Another possibility is multipartite viruses, where the genome is split into different segments, which can be transmitted separately, and where the different segments can be complementary or mutually dependent (Leeks et al., 2021, 2023; Lowen, 2018). The stability of this interaction could be compromised by oscillations in frequency, and so it would be extremely useful to examine what conditions prevent oscillations. Still, another possibility is to consider the coevolution of multiple social traits, such as rock–paper–scissors dynamics (Inglis et al., 2016; Kerr et al., 2002). In all these cases, it would be useful to examine the effects of population bottlenecks in structured or unstructured population.

## Supplementary Material

qrad032_suppl_Supplementary_Material

## Data Availability

All codes and data of this study are available at: https://github.com/mingpapilio/Codes_Oscillation.
