# Supplementary material for: Equilibria and oscillations in cheat–cooperator dynamics
Source: Evol Lett. 2023 Jul 22;7(5):339–50. doi: 10.1093/evlett/qrad032 (PMC10565900; doi:10.1093/evlett/qrad032)
Supplement: qrad032_suppl_Supplementary_Material [file qrad032_suppl_supplementary_material.pdf]

Equilibria and oscillations in cheat-cooperator dynamics

Supplementary Information

Ming Liu, Geoff Wild, and Stuart A. West

June 19, 2023

This document is the supplementary information for the **Equilibria and oscillations in cheat-cooperator dynamics** and contains four sections: The first section has the list of all parameters used in the model; the second section is a broader investigation for frequency and density dependence in scenario 1; the third section contains additional results and checking related to periodic population bottlenecks in scenario 2; the fourth section has the additional results related stochasticity in scenario 3.

Contents

1 General **2**
1.1 Table of all model parameters . . . 2

2 Frequency and density dependence (scenario 1) **3**
2.1 Dynamics of various weightings of density and frequency dependencies . . . 3
2.2 Analytical stability analysis . . . 3

3 Periodic population bottlenecks (scenario 2) **5**
3.1 Parameters in density and frequency dependence function . . . 5
3.2 ODE solver checking . . . 6
3.3 Intrinsic growth rate checking (r) . . . 7
3.4 Effects of the costs of cooperation (h) . . . 7
3.5 Relative fitness of cheats . . . 8
3.6 Effects of dilution ratio (D) . . . 10
3.7 Effects of growing time (T_{grow}) . . . 10
3.8 Effects of shape patameter (s_d, s_f) . . . 11
3.9 Effects of threshold parameter (t_d, t_f) . . . 13

4 Stochastic group formation (scenario 3) **15**
4.1 Simulation setup of scenario 3 . . . 15
4.2 Reducing stochasticity through increasing carrying capacity (K) . . . 16
4.3 Relative fitness of cheats on a board scale . . . 17
4.4 Effects of mixing coefficient (F) . . . 18
4.5 Effects of mixing coefficient when cheats are obligate (F, a or b= *1*) . . . 19

1 General

1.1 Table of all model parameters

Table S1: The list of parameters in the model. Sections column provides the relavent section numbers in this document for each parameters.

Name	Value	Description	Sections
**General variables used in all scenarios**			
r	2	Intrinsic growth rate	3.3
K	10	Carrying capacity (set to 100 in scenario 3)	4.2
h	4	Benefit coefficient of cheats	3.4
s_d	3	Shape coefficient of density dependence function	3.8
t_d	1.5	Threshold of density dependence function	3.9
s_f	10	Shape coefficient of frequency dependence function	3.8
t_f	0.5	Threshold of frequency dependence function	3.9
**For growth cycles (scenario 2& 3)**			
D	10	Dilution ratio (set to 5 in scenario 3)	3.6
T_{grow}	4	Duration of growth phase in each growth cycle	3.7
**For stochasticity (scenario 3)**			
F	1.3	Mixing coefficient	4.4, 4.5
M	4000	Number of subpopulation (social groups)	Fig. 4

2 Frequency and density dependence (scenario 1)

2.1 Dynamics of various weightings of density and frequency dependencies

In Fig. 2 of the main text, we showed the population dynamics quickly become static after carrying capacity has been reached, we tested whether the result was just a special case in this section. As shown in Fig. S1, we found the same dynamics across all combinations of weighting coefficients. These results suggest a relatively static dynamic is prevalent.

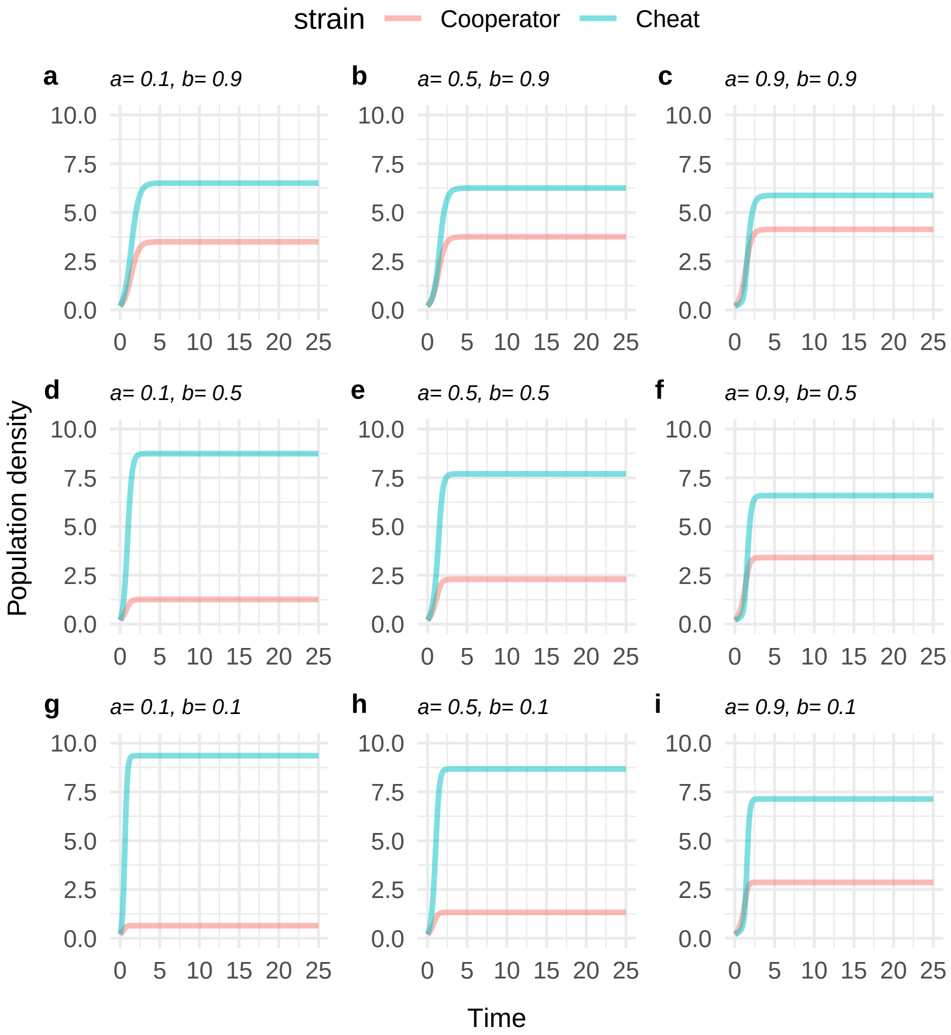

Figure S1: Population dynamics across various combinations of weightings in the baseline setting.

2.2 Analytical stability analysis

We re-write Eq (2) of the main text as

$$\begin{aligned} N'_{\rm co} &= F(N_{\rm co} + \alpha\, N_{\rm ch}; K)\, N_{\rm co} \\ N'_{\rm ch} &= h\, G(N_{\rm co}; a, s_d, t_d)\, G(\tfrac{N_{\rm co}}{N_{\rm co}+N_{\rm ch}}; b, s_f, t_f)\, F(\alpha\, N_{\rm co} + N_{\rm ch}; K)\, N_{\rm ch} \end{aligned} \tag{S1}$$

where

$$F(N;K) = 1 - \frac{N}{K}, \quad \text{and} \quad G(N;a,s,t) = 1 - a + a\frac{1}{1+\exp\{-s(N-t)\}} \tag{S2}$$

and $0 < \alpha \leq 1$ is constant.

If α is strictly less than 1, then

$$N^*_{\rm co} = N^*_{\rm ch} = \frac{K}{1+\alpha} \tag{S3}$$

is a unique, positive equilibrium; it is locally asymptotically stable when all eigenvalues of

$$J = \begin{bmatrix} F'(K)\, N^*_{\rm co} & \alpha\, F'(K)\, N^*_{\rm co} \\ \alpha\, h\, G(N^*_{\rm co})\, G(\frac{N^*_{\rm co}}{N^*_{\rm co}+N^*_{\rm ch}})\, F'(K)\, N^*_{\rm ch} & h\, G(N^*_{\rm co})\, G(\frac{N^*_{\rm co}}{N^*_{\rm co}+N^*_{\rm ch}})\, F'(K)\, N^*_{\rm ch} \end{bmatrix} \tag{S4}$$

have negative real part (parameters of G and F have been omitted, for simplicity). The Routh-Hurwitz criteria for two-dimensional systems tell us that the eigenvalue condition on J is satisfied when both its trace is negative and its determinant is positive. Direct calculation shows

$$\text{trace}\, J = F'(K) \left(N^*_{\rm co} + h\, G(N^*_{\rm co})\, G\left(\frac{N^*_{\rm co}}{N^*_{\rm co} + N^*_{\rm ch}} \right) N^*_{\rm ch} \right) < 0 \tag{S5}$$

and

$$\det J = h\, G(N^*_{\rm co})\, G\left(\frac{N^*_{\rm co}}{N^*_{\rm co} + N^*_{\rm ch}} \right) (F'(K))^2\, N^*_{\rm ch}\, N^*_{\rm co}\, (1-\alpha^2) > 0 \tag{S6}$$

which implies the equilibrium is locally asymptotic stable. A sketch of the phase plane shows not only that the equilibrium is globally stable but also that periodic (oscillating) trajectories do not occur (Fig S). We formalize the line of reasoning, here, using Dulac's criterion. Define $H(N_{\rm co}, N_{\rm ch})$ as $\frac{1}{N_{\rm co}\, N_{\rm ch}}$ and consider the vector field, V, defined (strictly inside the first quadrant of the plane) as

$$V(N_{\rm co}, N_{\rm ch}) = \left(\frac{F(N_{\rm co} + \alpha N_{\rm ch})}{N_{\rm ch}},\ h\, G(N_{\rm co})\, G(\tfrac{N_{\rm co}}{N_{\rm co}+N_{\rm ch}})\, \frac{F(N_{\rm ch} + \alpha N_{\rm co})}{N_{\rm co}} \right) \tag{S7}$$

where, again, we have omitted the parameters of F and G for simplicity. We calculate the divergence of V to be

$$\begin{aligned} \nabla \cdot V = \frac{F'(N_{\rm co} + \alpha N_{\rm ch})}{N_{\rm ch}} &+ h\, G(N_{\rm co})\, G'(\tfrac{N_{\rm co}}{N_{\rm co}+N_{\rm ch}})\, (-1) \frac{N_{\rm co}}{(N_{\rm co}+N_{\rm ch})^2}\, \frac{F(N_{\rm ch} + \alpha N_{\rm co})}{N_{\rm co}} \\ &+ h\, G(N_{\rm co})\, G(\tfrac{N_{\rm co}}{N_{\rm co}+N_{\rm ch}})\, \frac{F'(N_{\rm ch} + \alpha N_{\rm co})}{N_{\rm co}}. \end{aligned} \tag{S8}$$

Because $F' < 0$ and $G' > 0$ we can be sure that $\nabla \cdot V < 0$. Following Dulac's criterion, then, we formally rule out the existence of periodic solutions to Eq (S1), because $\nabla \cdot V$ neither changes sign nor is identically zero. Because trajectories in the planar system, represented by Eq (S1), are bounded (Fig. S2), the Poincaré-Bendixson Theorem tells us that they must either tend to an equilibrium or a periodic orbit thereof. Given that we have ruled out the latter, it must be true that solutions tend to the only equilibrium we have identified inside the first quadrant.

If $\alpha = 1$, then we can replace the variables $N_{\rm co}$ and $N_{\rm ch}$ with $N = N_{\rm co} + N_{\rm ch}$ and $P = \frac{N_{\rm co}}{N}$. With these new variables the dynamics become

$$\begin{aligned} N' &= (P + h\, G(PN)\, G(P)\, (1-P))\, F(N)\, N, \\ P' &= P(1-P)\, (1 - h\, G(PN)\, G(P)\,)\ F(N). \end{aligned} \tag{S9}$$

As an aside, we note that the rate of change in the proportion of cooperators is the product of the trait variance (namely, $P(1-P)$) and the change in fitness experienced by an individual who switches from cheating to cooperation (namely, $F(N) - h\, G(PN)\, G(P)\, F(N)$). The sign of N' is solely determined by $F(N)$. Therefore, over time, N converges monotonically to K (the line $N_{\rm co} + N_{\rm ch} = K$ in Fig 2c). The sign of P' is also solely determined by $F(N)$, and so the evolution of P will follow that of N (P increases when N increases and decreases when N decreases). It follows that P will also tend to some equilibrium in a monotonic fashion. Overall, we rule out the existence of periodic solutions when $\alpha = 1$.

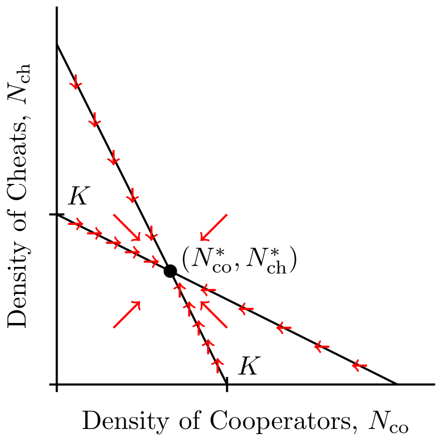

Figure S2: A sketch of the phase plane associated with Eq (S1). Red arrows show the direction followed by trajectories over time. Solid lines show the set of states for which $N'_{co}=0$ and $N'_{ch}=0$, respectively. For the former set, trajectories are instantaneously moving in a purely vertical direction; for the latter set, trajectories are instantaneously moving in a purely horizontal direction. Solid lines intersect at the equilibrium $(N*_{co}, N*_{ch})$, and this equilibrium is asymptotically stable.

3 Periodic population bottlenecks (scenario 2)

3.1 Parameters in density and frequency dependence function

Our density and frequency dependence functions share the same the basic structure of

$$f(x) = h(1 - a + \frac{a}{1 + e^{-s(x-t)}}), \tag{S10}$$

where x is density (or frequency) of cooperators in the population, h is the coefficient of relative growth advantage of cheats, which is a proxy for the costs of cooperation, $a (or b)$ is the weighting coefficient of the dependence, s is the shape coefficient adjusting the steepness, and t is the threshold, in which the steepest change in response takes place. The effects of each parameter is shown in Fig. S3: increasing a results in a lower response at low cooperator density (or frequency; Fig. S3a), increasing h results in greater response after threshold is passed (Fig. S3b); increasing s results in steeper response, making cheat's dependence more restricted to a specific range (Fig. S3c); increasing t horizontally shifts the response of cheats (Fig. S3d).

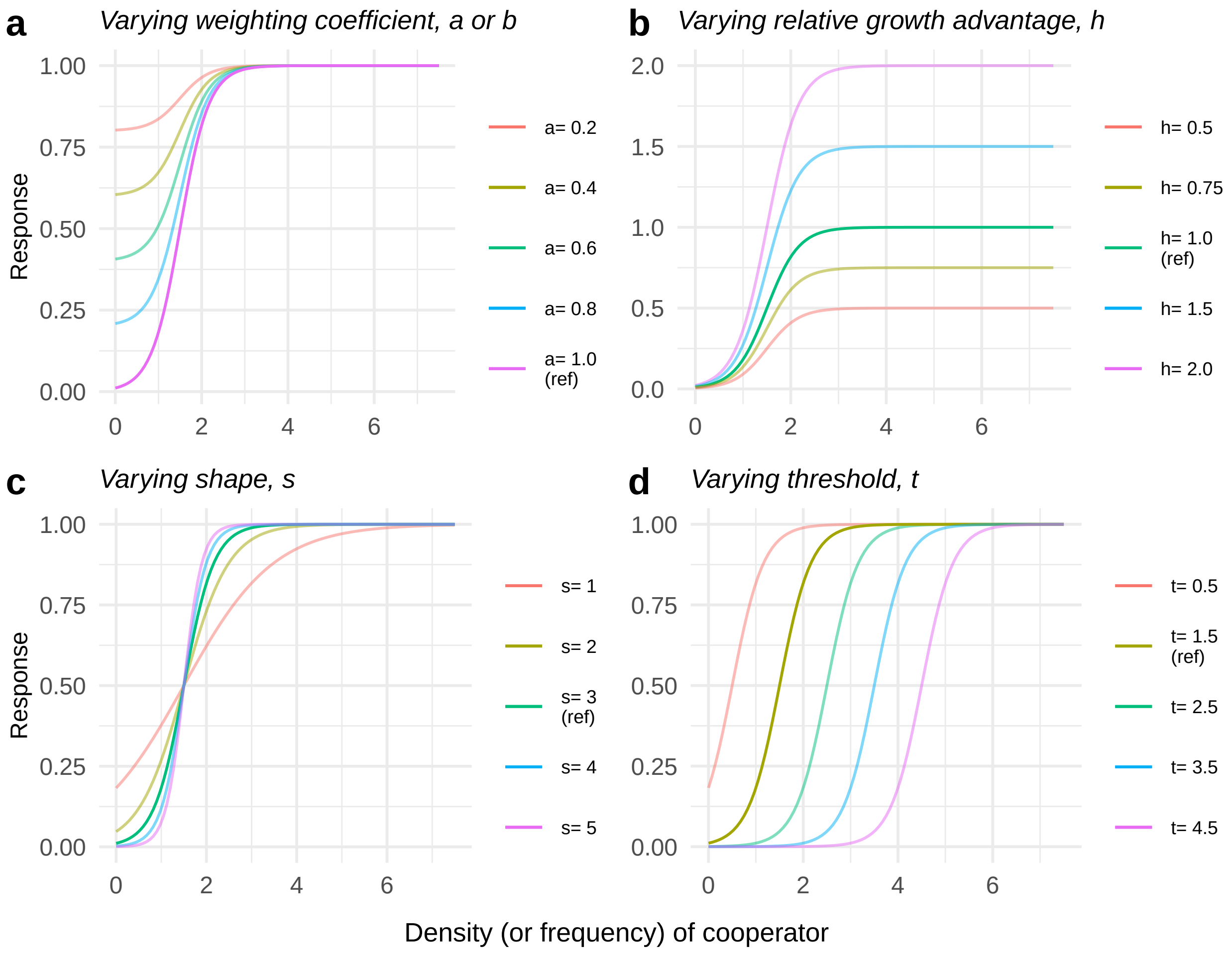

Figure S3: Parameters of logistic function used in density and frequency dependence terms.

3.2 ODE solver checking

We set up the ODE solver (the standard package in Python3: scipy.integrate.odeint) to numerically track the population dynamics. The time in each iteration of the solver is adjustable that bigger leap means less precision but less calculation time. Thus, we checked the precision setting to make sure we are not generating numerical artifacts in our result. As shown in Fig. S4, the dynamics in all panels are close to identical, suggesting all settings have reasonable precision. Thus, we use 20 iterations per time unit for all analysis (Fig. S4b).

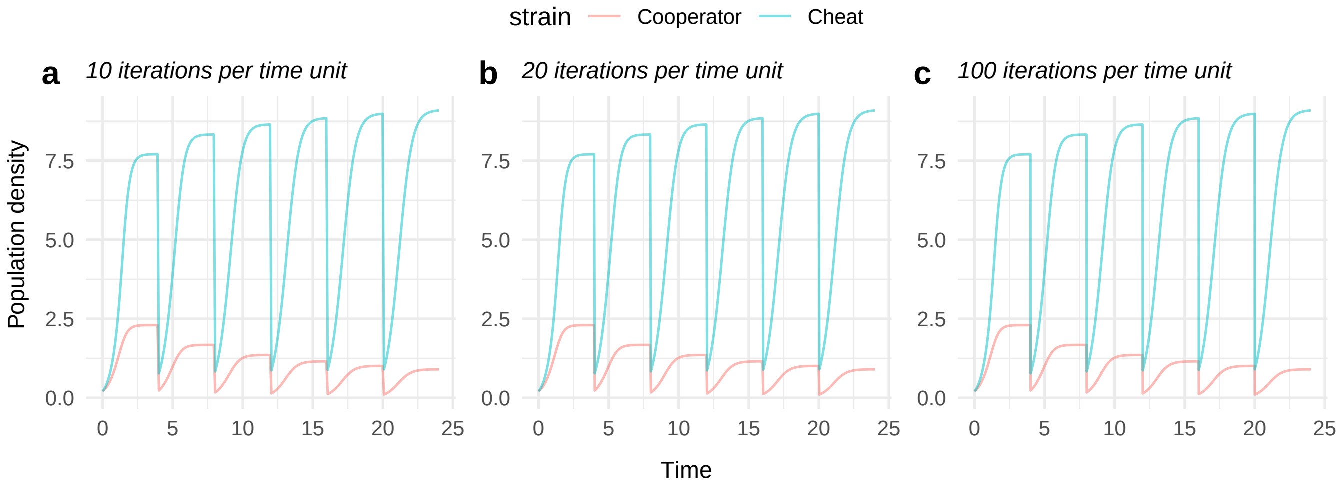

Figure S4: Time series of population dynamics under different precisions (a=*0.5*, b=*0.5*).

3.3 Intrinsic growth rate checking (r)

We then checked the effects of intrinsic growth rate to make sure the oscillation is not an artifact from special combinations of growth cycle (i.e., periodic population bottleneck) and growth rate (r). We found the pattern of oscillation is robust as long as $r > 1.5$ in Fig. S5. Hence, we have been using $r = 2$ to make sure the results are good representations for most ranges of the intrinsic growth rate.

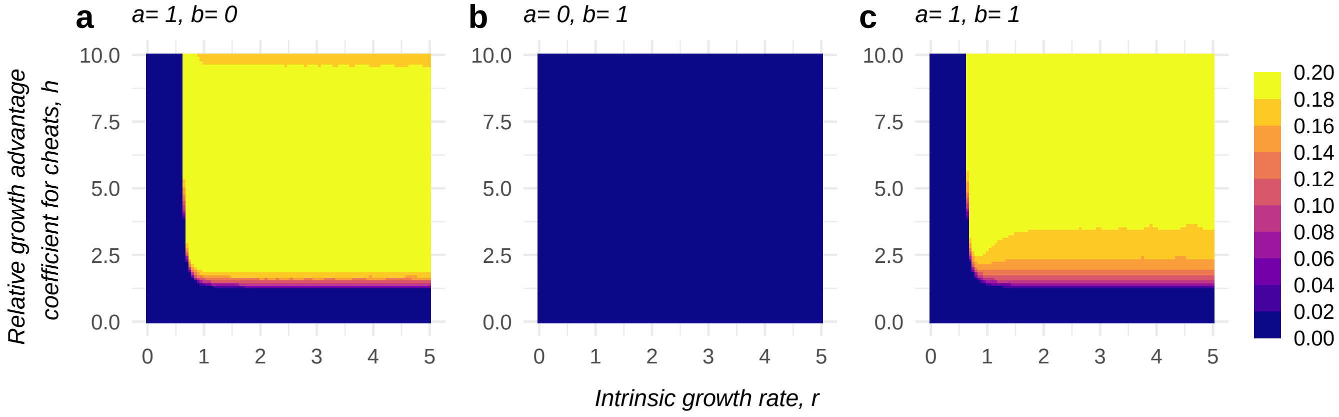

Figure S5: Amplitude of oscillation in proportion of cheats across various intrinsic growth rates, and relative growth advantage coefficient of cheat. Brighter colour indicates larger oscillation and the amplitude is calculated from the maximal difference in proportion of cheats in each growth cycle after the dynamics become stabilised.

3.4 Effects of the costs of cooperation (h)

We use the coefficient of relative growth advantage of cheats, h, as a proxy for the cost of performing cooperation and we found as h increases, a region of no oscillation expands (arrows in Fig. S6b-d). The region is where both weightings are small, meaning cheats do not depend as much on the density or frequency (proportion) of cooperators. The underlying reason is as h is at a larger value, cheats can out-compete cooperators in a wider range of parameters. In contrast, the result when weighting is at 1 does not change with h, as cheats cannot eliminate cooperators when there is complete dependence in one of the two weightings. Lastly, we found no oscillation at all when $h = 1$ as the fitness of cheats never exceeds cooperator in such setting (i.e., cheats are eliminated).

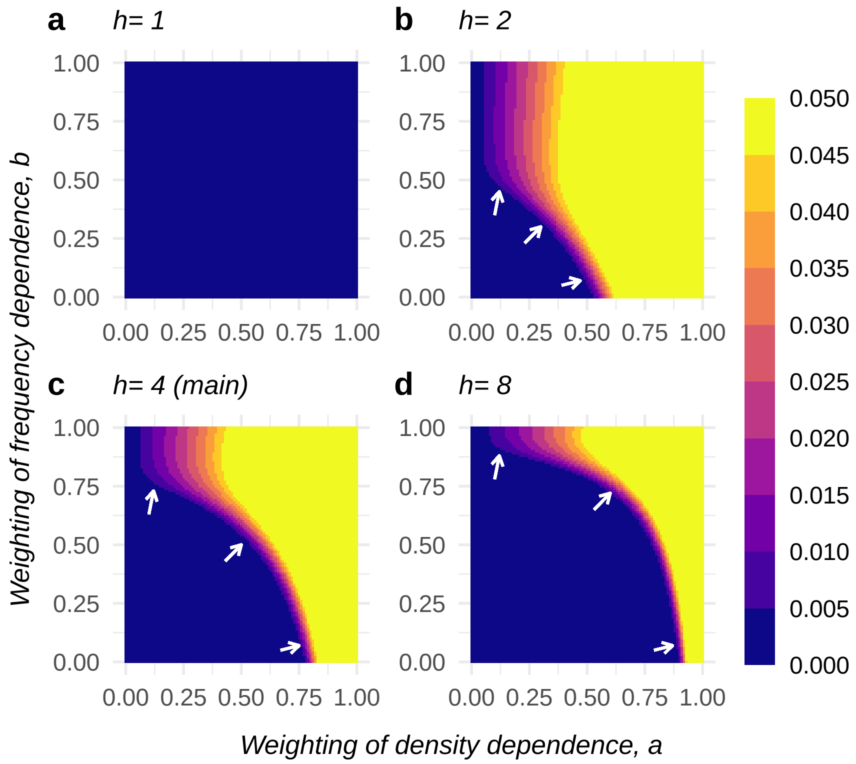

Figure S6: Amplitude of oscillation across various relative growth advantage coefficient for cheats. Higher h means higher benefit of cheats and brighter colour indicates larger oscillation.

3.5 Relative fitness of cheats

One important factor that determines whether cheats or cooperators win is the relative fitness, which is defined as

$$w_{rel,ch} = \frac{w_{ch}}{p_{ch}w_{ch} + (1 - p_{ch})w_{co}}, \tag{S11}$$

where p_{ch} is the proportion of cheats in the population, w_{ch} is the per capita growth rate of cheats, $\frac{dN_{ch}}{N_{ch}dt}$, and w_{co} is the per capita growth rate of cooperator, $\frac{dN_{co}}{N_{co}dt}$. When $w_{rel,ch} > 1$, cheats can outgrow cooperators and potentially eliminate cooperators; when $w_{rel,ch} < 1$, cooperators grow better than cheats and can potentially eliminate cheats.

We looked into two quantities of relative fitness: (1) the maximal difference within each growth cycle, and (2) the average relative fitness over a single growth cycle. We found the pattern in differences is similar to the pattern in oscillation amplitudes (Fig. S7 and Fig. S6). The only exception is when $h = 1$ because the brighter colors in Fig. S7a mean cheats are much worse than cooperators, whereas brighter colors other panels mean cheats are doing better than cooperators. This explanation is supported by the average relative fitness (Fig. S8), that we only found relative fitness lower than 1 when $h = 1$. In addition, we found the region of $w_{rel,ch} \approx 1$ expands as h increases because relative fitness is 1 for cheats when there is no cooperator in the population (arrows in Fig. S8b-d).

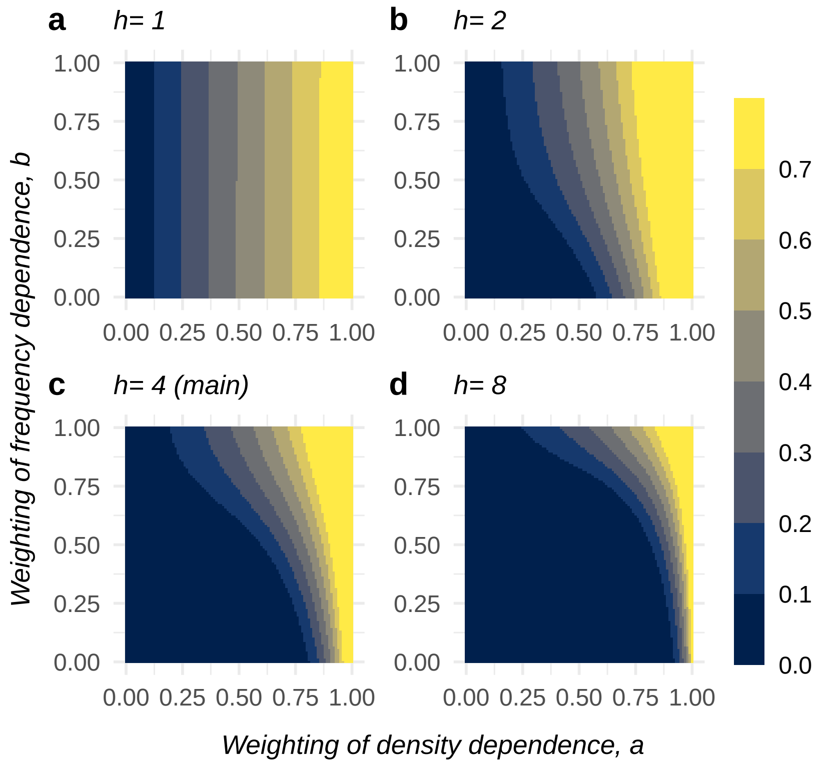

Figure S7: Maximum difference in relative fitness of cheats in each growth cycle.

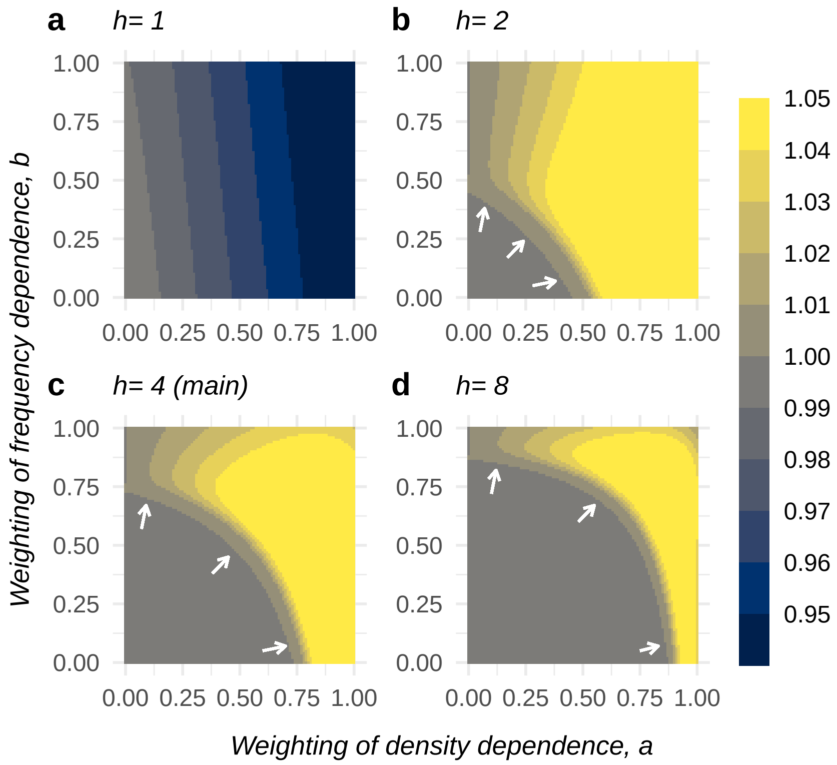

Figure S8: Average of relative fitness of cheats in each growth cycle.

3.6 Effects of dilution ratio (D)

The next factor we investigated is the dilution ratio, which controls the size of population bottleneck between growth cycles. Intuitively, we found when D is too low, there is almost no oscillation (Fig. S9a-b), while the pattern roughly stays the same when D is large (Fig. S9c-d). The reason is when there is insufficient dilution would prevent cooperators from recovering their proportion in the population and eventually lead to extinction (see also Fig. 6a-e in main text).

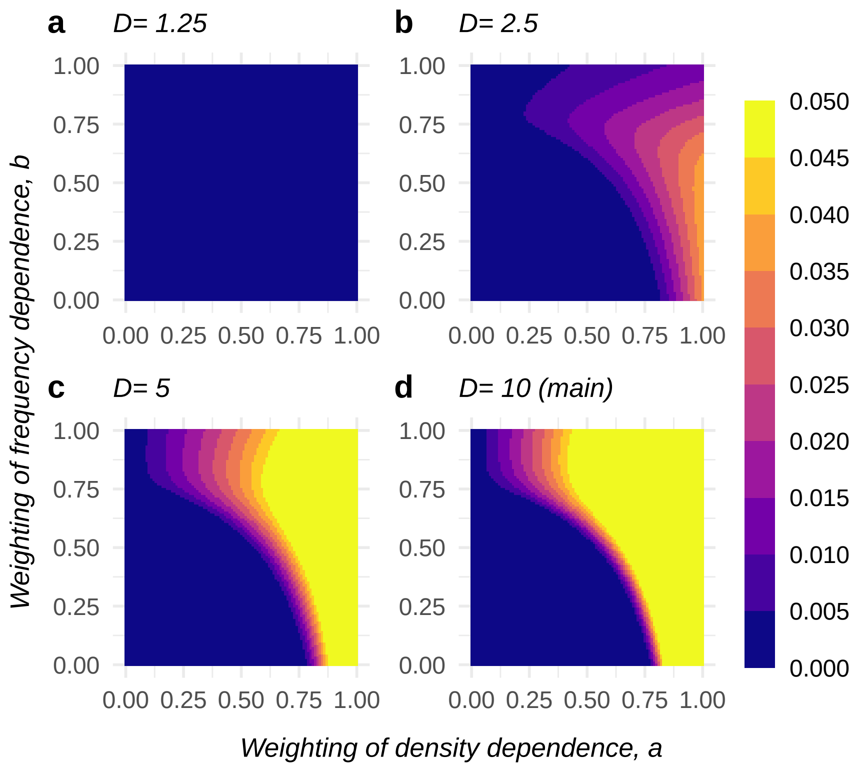

Figure S9: Amplitude of oscillation across various dilution ratios.

3.7 Effects of growing time (T_{grow})

Another growth cycle-related factor is the duration of growth time in each growth cycle, which determines whether dilution takes place at lag, exponential, or stationary phase of the growth curve. We found once T_{grow} is greater than 2, the results is identical regardless of the duration of growing time (Fig. S10b-d). This result is also easy to explain as cheats would not have time to grow and increase their proportion if growing time is too short (Fig. S9a). Yet, once cheats have chances to gain proportion, there is little difference in the results because both strains would saturate in densities as growing time increases.

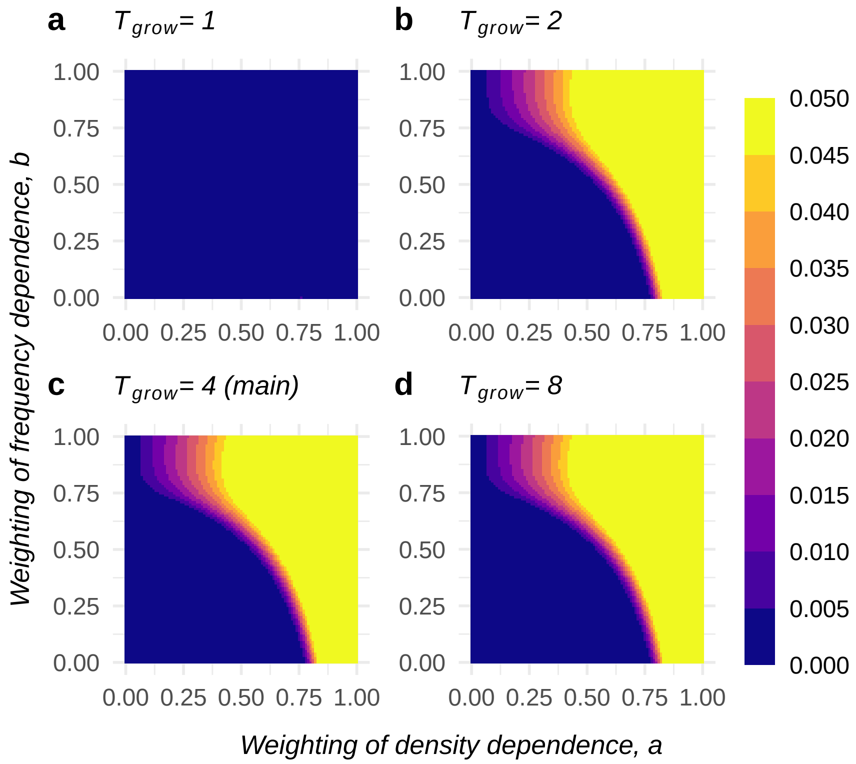

Figure S10: Amplitude of oscillation across various duration of growth phase.

3.8 Effects of shape patameter (s_d, s_f)

Besides the settings in growth cycle, another important aspect of the model is the parameters controlling the density and frequency dependence. We began from the shape parameters, s_d and s_f, where larger s means steeper response (Fig. S3c). We found increasing s_d always help oscillation as yellow area consistently increases as s_d increases (Fig. S11). On the contrary, the effect of s_f is inconsistent as it helps oscillation when $s_f < 10$ but somewhat hinders oscillation when $s_f > 10$ (Fig. S12). This discrepancy happens because (i) density can fluctuate through dilution processes between growth cycle while frequency does not change through dilution, and (ii) increasing s would narrow the range where response can be different. In other words, the fitness landscape becomes more binary when s increases, while density dependence still have the drive to bounce between lower and higher responses (through dilution), frequency dependence completely relied on relative fitness to oscillate and it is harder to move around when the response is too steep. One final note is we set s_d and s_f to different values because the range of density is larger than the range of frequency, which is confined in $\{0, 1\}$.

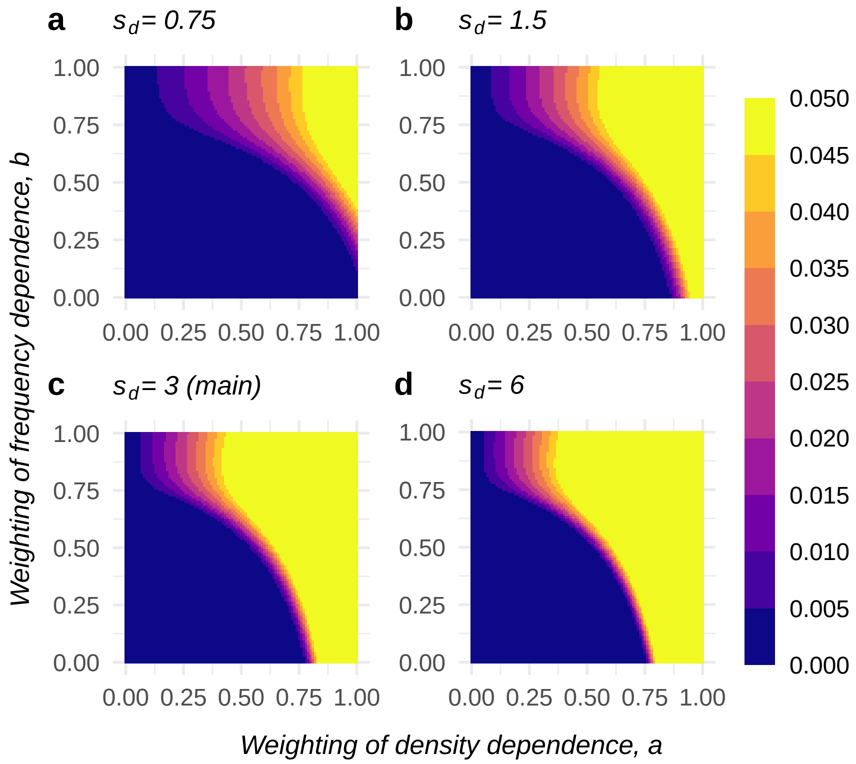

Figure S11: Amplitude of oscillation across various shape parameter of density dependence.

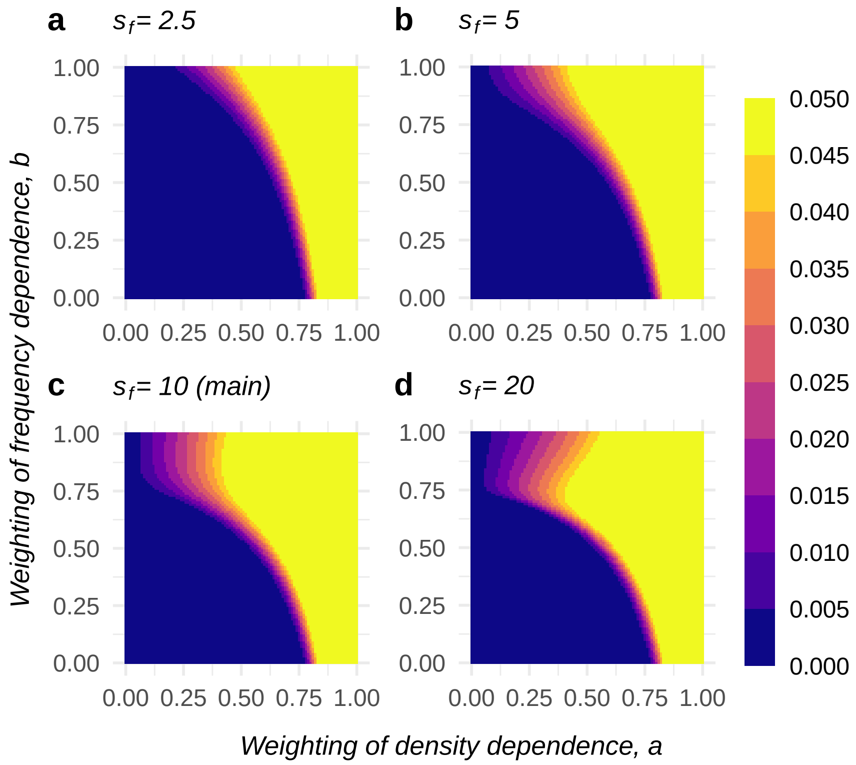

Figure S12: Amplitude of oscillation across various shape parameter of frequency dependence.

3.9 Effects of threshold parameter (t_d, t_f)

The last parameter we examined in scenario 2 is the threshold of the response, t_d and t_f, which horizontally move the response to different ranges (Fig. S3d). We found changing thresholds result in diverse outcomes. Specifically, increasing t_d initially increases the region of oscillation, but when $t_d > 1.5$, increasing threshold hampers oscillations (Fig. S13). This is likely because when threshold is too high or too low, the majority of time in the growth cycle would favour one of the two strains (favouring cheats when threshold is low and cooperators when threshold is high), and prevent oscillation. In one extreme case, when $t_d = 6$ and $a > 0.9$, there is no oscillation as cooperator density hardly exceeds 6 and cheats cannot grow (Fig. S13d) On the other hand, the pattern of changing thresholds in frequency is similar to changing shapes in frequency dependence (Fig. S14).

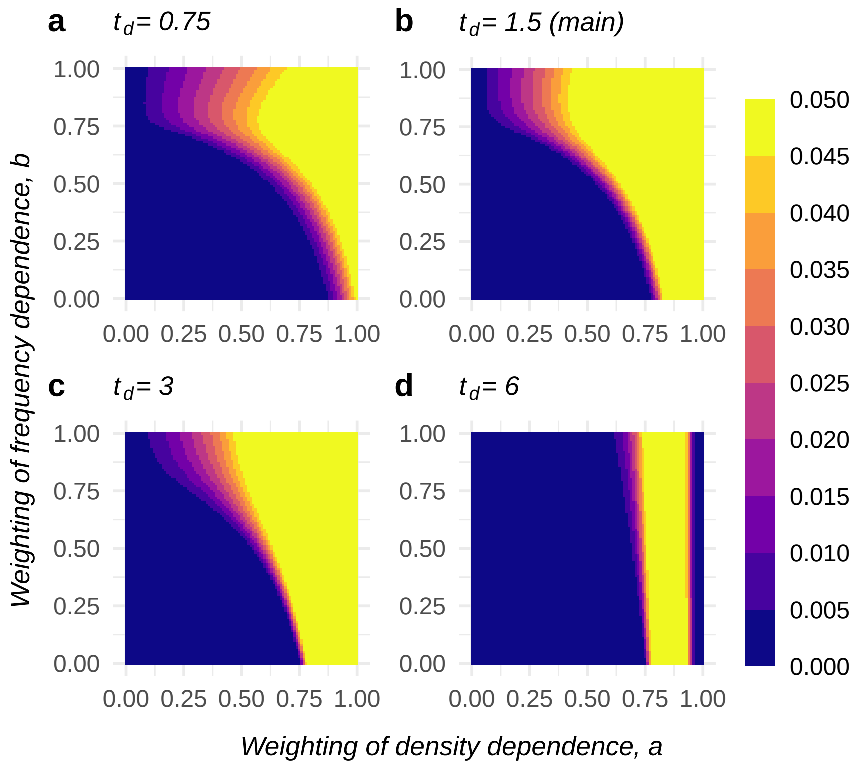

Figure S13: Amplitude of oscillation across various threshold of density dependence.

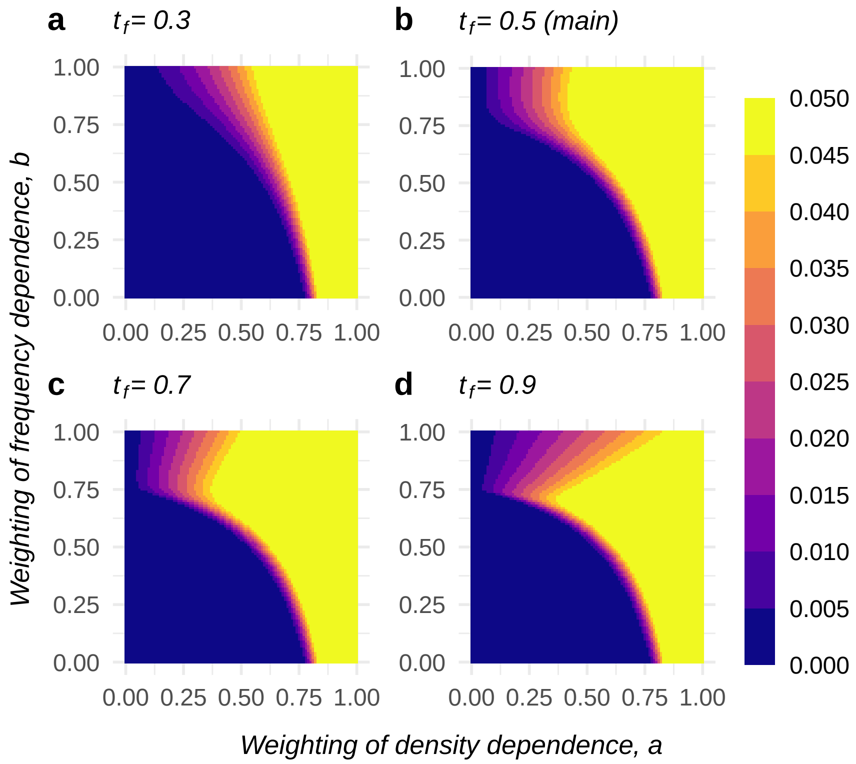

Figure S14: Amplitude of oscillation across various threshold of frequency dependence.

4 Stochastic group formation (scenario 3)

4.1 Simulation setup of scenario 3

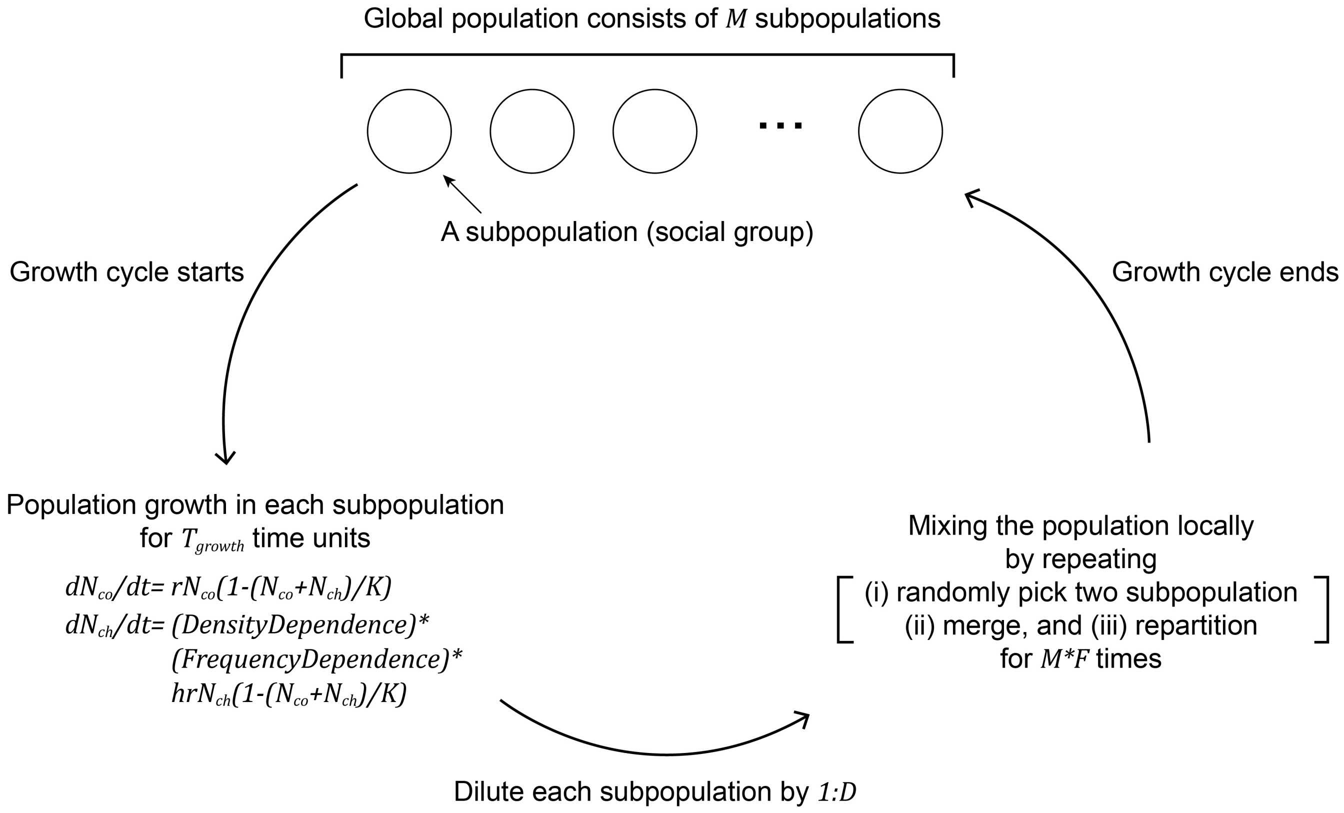

Figure S15: Schematic view of simulation steps in scenario 3, where the simulation codes are modified from Muzuuchi et al, 2022. Following their design, we convert the densities of cheats and cooperators to integer after dilution, and we use binomial process with equal probabilities to re-disperse each unit of cheats and cooperators during repartitioning process. Both processes can help generate stochasticity to the system.

4.2 Reducing stochasticity through increasing carrying capacity (K)

In addition to Fig. 4 in main text, an alternative way to manipulate stochasticity is to increase the population size within each subpopulation. This can be done through increasing carrying capacity, K. Nevertheless, the duration of growth cycle and the threshold of dependence have to change because the density of the two strain is going to be at a different level (see figure caption of Fig. S16 for more information). We found the amplitude of oscillation decreases as carrying capacity increases (Fig. S16g-i) and the result is consistent with Fig. 4.

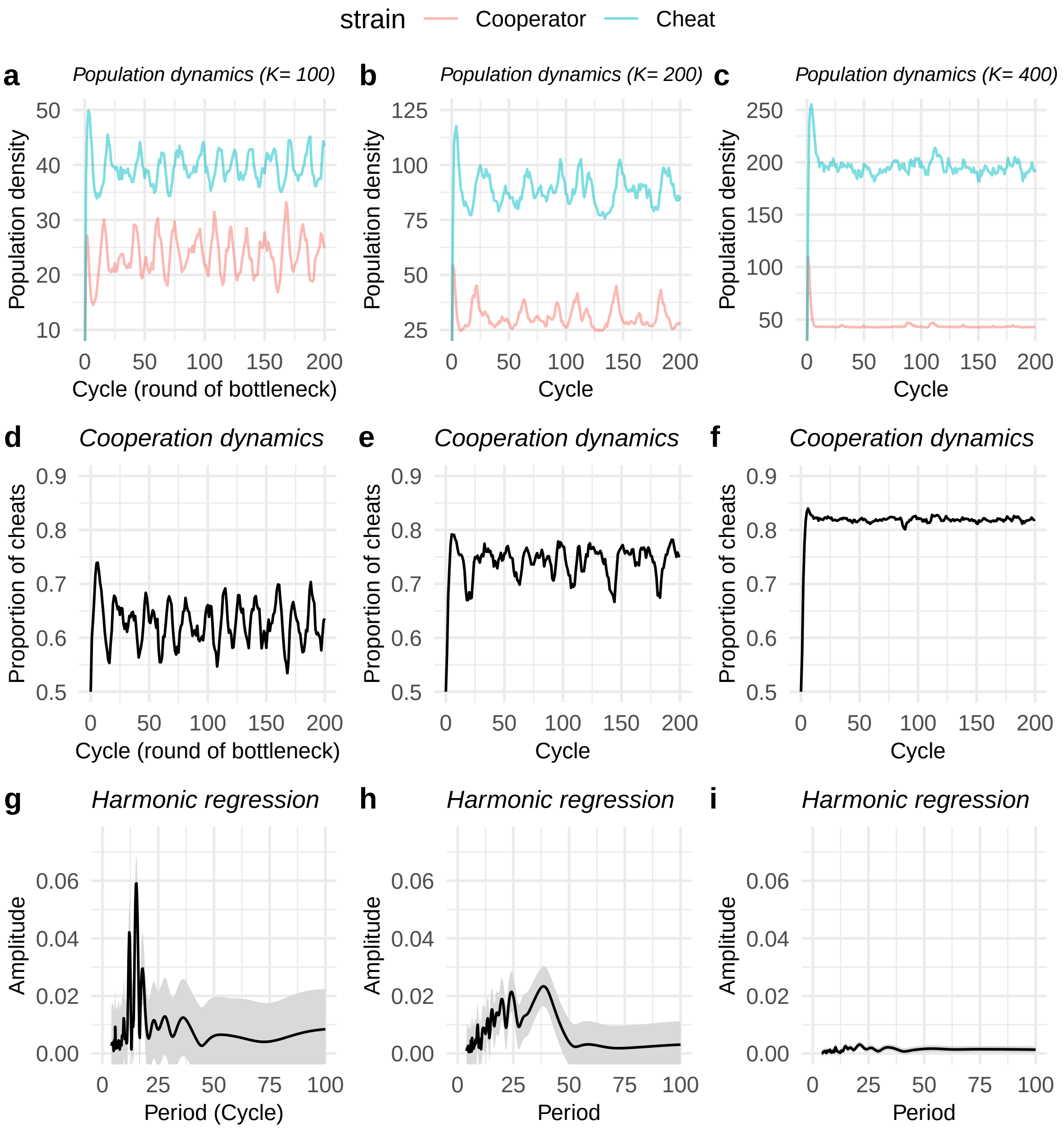

Figure S16: Increasing carrying capacity within each subpopulation decreases stochasticity and produce smaller oscillation in proportion of cheats. Growth cycle and threshold are changed to different values to allow population having sufficient growing time (T_{grow} is 8 and t_d is 6 for $K = 200$; T_{grow} is 16 and t_d is 9 for $K = 400$; $a = 1$ and $b = 0$).

4.3 Relative fitness of cheats on a board scale

In Fig. S17, we found the average relative fitness of cheats is above 1 but close to 1 at the regions where oscillating dynamics take place (Fig. 5). Together with Fig. 5, these results suggest oscillation happens at the parameter space (1) where cheats are slightly fitter than cooperators and (2) cheats' fitness is highly variable between subpopulations.

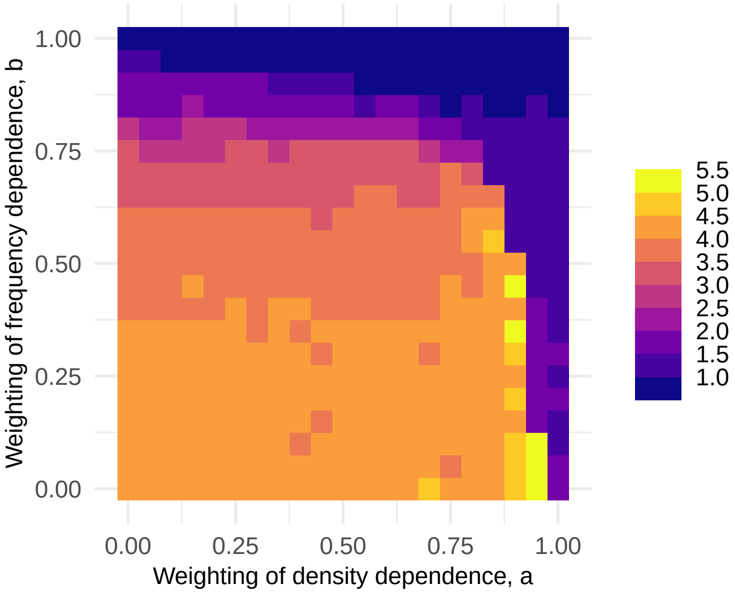

Figure S17: Relative fitness of cheats. Brighter color means cheats having greater fitness over cooperators. Relative fitness is calculated at subpopulation level, based on the proportion of cheats within each subpopulation at the beginning of focal growth cycle. The absolute fitness used for relative fitness is approximated from per capita growth rate, the change in cheat or cooperator density within subpopulation, divided by T_{grow} and the initial densities. We then averaged the relative fitness over all subpopulations and time, and rescaled the relative fitness by dividing it with the relative fitness of cooperators because the raw values are deflated by empty subpopulations. Each grid is the average of 10 repeated simulations. All simulations last 200 growth cycles and data was collected from cycle 51 to 200. The number of subpopulations is set to 4000.

4.4 Effects of mixing coefficient (F)

We examined the effects of mixing coefficient as it can potentially change the spatial structure of the entire population and help the spread of cheats or cooperators. We found that greater mixing produces a less periodic dynamic - as the mixing coefficient (F) increases, the peak signal in harmonic regression become smaller, while the non-peak signals become larger (Fig. S18d& e). This is because faster mixing makes the spread of cheats and cooperators faster, resulting in less periodic dynamics (Fig. S18c& f).

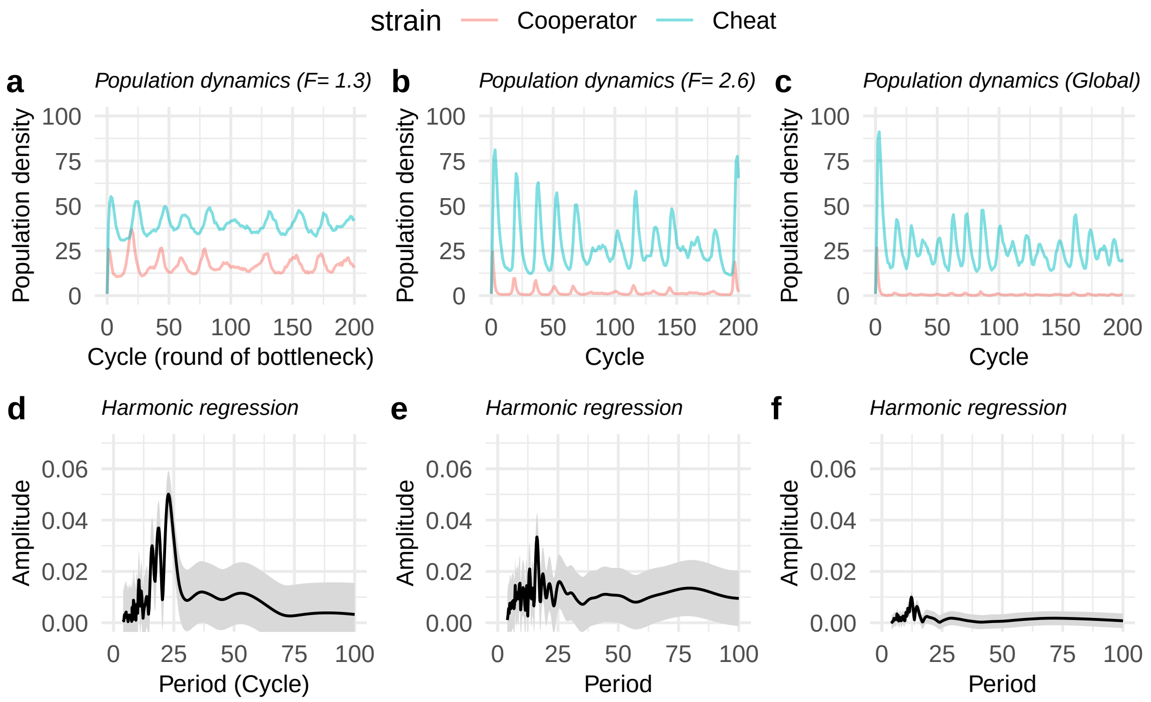

Figure S18: Local mixing is crucial for oscillation across growth cycles. (a-c) Population dynamics under default mixing (a, F=*1.3*), twice times of mixing (b, F=*2.6*), and global mixing after the dilution in each growth cycle. In global mixing setting, subpopulations are re-initialised through Poisson sampling after summing the density of cheat and cooperator and after periodic population bottlenecks, so stochasticity is not reduced to zero. (d-f) The corresponding Harmonic regression on proportion of cheats in each setting. Density and frequency dependence are set to the combination where largest coefficient of variation was observed in Fig. 5 (a=*0.9*, b=*0.25*).

4.5 Effects of mixing coefficient when cheats are obligate (F, a or b= 1)

In addition to Fig. S18, where cheats are facultative that they can grow in the absence of cooperator, we found some interesting result when cheat is obligate. Specifically, we found increasing mixing results in lower proportion of cheats (Fig. S19). Further, increasing mixing produces more frequent oscillations as the peak in harmonic regression moves to the left when F is larger. This is because of the complication that cheats are obligate cheats in this case and cannot grow when cooperator is absent in the group These results suggest mixing is a crucial factor of our model and it can determine the extent of periodicity and noise in the dynamics.

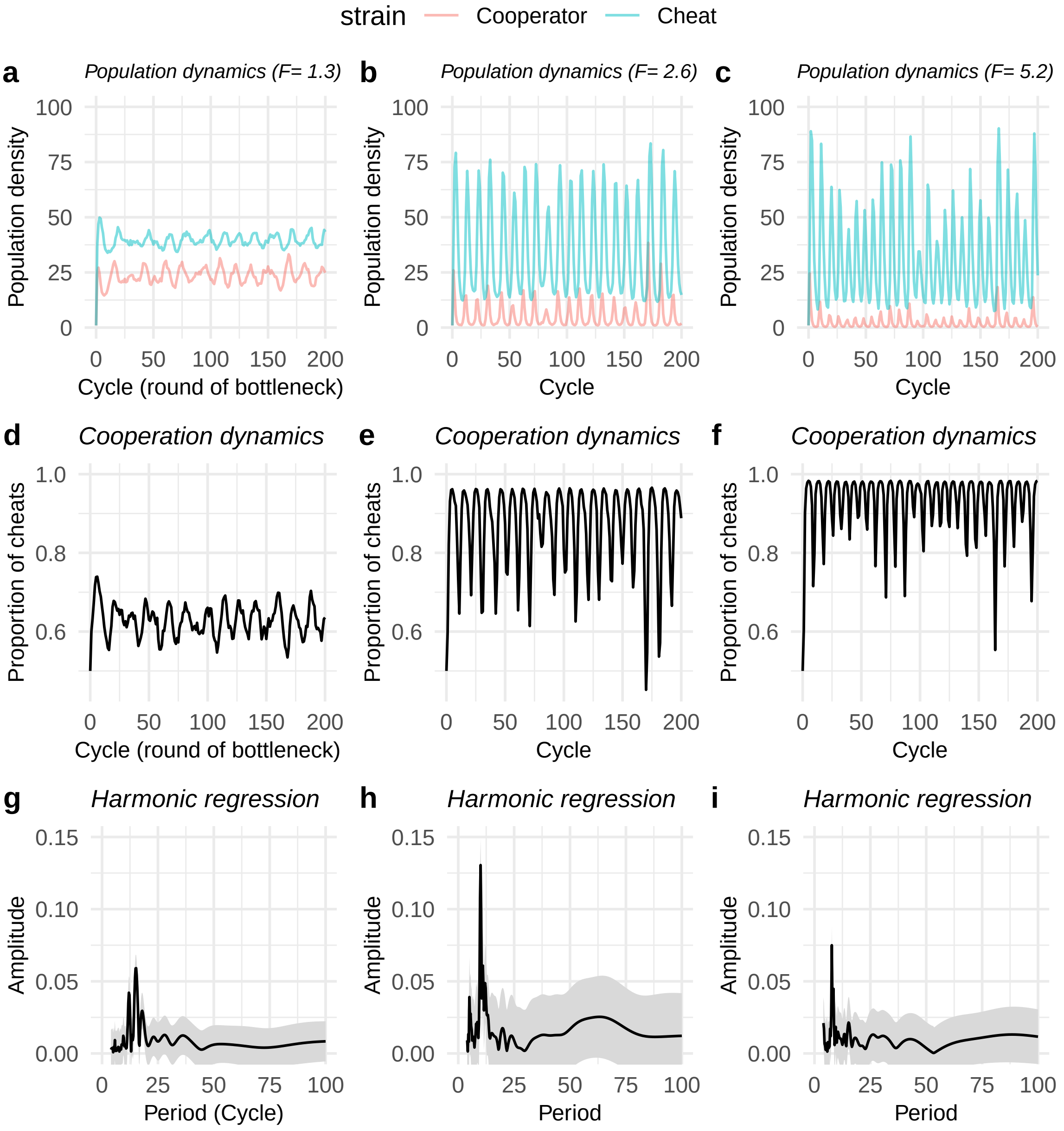

Figure S19: Increasing mixing coefficient results in more frequent oscillation when cheats can only grow when cooperator is present (a=1, b=0).
